# A previously unknown Argonaute 2 variant positively modulates the viability of melanoma cells

**DOI:** 10.1007/s00018-022-04496-8

**Published:** 2022-08-09

**Authors:** Lisa Linck-Paulus, Tina Meißgeier, Katharina Pieger, Anselm H. C. Horn, Alexander Matthies, Stefan Fischer, Gunter Meister, Heinrich Sticht, Melanie Kappelmann-Fenzl, Anja K. Bosserhoff

**Affiliations:** 1 Institute of Biochemistry, Friedrich-Alexander-Universität Erlangen-Nürnberg (FAU), 91054 Erlangen, Germany; 2 Erlangen National High Performance Computing Center (NHR@FAU), Friedrich-Alexander-Universität Erlangen-Nürnberg (FAU), 91058 Erlangen, Germany; 3 Faculty of Computer Science, University of Applied Science, 94469 Deggendorf, Germany; 4 Regensburg Center for Biochemistry (RCB), University of Regensburg, 93053 Regensburg, Germany

**Keywords:** Melanoma, Argonaute 2, miRNAs, Alternative splicing

## Abstract

In malignant melanoma, a highly aggressive form of skin cancer, many microRNAs are aberrantly expressed contributing to tumorigenesis and progression. Further, deregulation of microRNA processing enzymes, like the miRNA-binding protein Argonaute 2, significantly impacts microRNA function. This study characterizes a novel splice variant of Argonaut 2, AGO2-ex1/3. AGO2-ex1/3 is substantially expressed in different melanoma cell lines and patient-derived tissue samples. It is a mature mRNA, which is translated into an N-terminally truncated Argonaute 2 protein form. Molecular dynamics simulations show that the PAZ, MID, and PIWI domain largely retain their structure in AGO2-ex1/3 and that the truncation of the N-terminus leads to an increased interdomain flexibility. Expression of AGO2-ex1/3 provides a survival advantage for melanoma cells while the knockdown causes significantly reduced proliferation and increases apoptosis. RNA-sequencing revealed that in cells lacking AGO2-ex1/3 expression many miRNA target genes are deregulated, implicating a considerable role of AGO2-ex1/3 for miRNA function. This study inaugurates insights into an important role of a so far unknown splice variant of Argonaute 2 for the miRNA pathway as well as the mechanisms which drive growth and survival of melanoma cells. This knowledge provides the basis for potential new promising therapeutic targets focusing on small RNA-mediated gene regulation in melanoma.

**Supplementary Information:**

The online version contains supplementary material available at 10.1007/s00018-022-04496-8.

## Introduction

Malignant melanoma is one of the most frequent solid tumor types and contributes to 90% of skin cancer-related mortality [1]. It is characterized by a still steadily increasing incidence and a high metastasis rate. Despite major advances in targeted therapy and immunotherapy in recent years, advanced metastatic melanoma is still not fully curable [2]. Therefore, it is essential to develop new therapeutic approaches based on a better understanding of the detailed molecular mechanisms leading to melanoma development and progression. Post-transcriptional gene regulation by small, non-coding RNAs, so called microRNAs (miRNAs), plays a pivotal role in all cellular processes associated with tumorigenesis like proliferation, migration, invasion or apoptosis [3]. MicroRNAs are part of the RNA induced silencing complex (RISC) together with Argonaute (AGO) proteins, which catalyze translational repression and degradation or storage of complementary target mRNAs [4]. MRNA cleavage only occurs when the AGO2-associated miRNA is perfectly complementary to the target. For translational repression, partial base pairing between miRNA and the target is sufficient. Human cells express four different AGO proteins. Whereas AGO1, 3 and 4 mostly act via recruitment of cellular factors for degradation of the bound mRNA, AGO2 has catalytic activity and can directly cleave the target mRNA via hydrolysis [5, 6].

In melanoma, the expression of many miRNAs is deregulated and affects important tumorigenic processes [7–9]. Interestingly, a majority of miRNAs is upregulated in melanoma cells compared to normal human epidermal melanocytes (NHEM) [7, 10]. This stands in contrast to other tumor types, where usually a loss or reduction of miRNA expression is observed [11, 12]. The cause of this melanoma unique miRNA upregulation has not yet been determined.

Further, not only deregulation of miRNAs can induce tumor development. It is known from many cancer types that deregulation of several proteins in the miRNA processing cascade is associated with tumor development and progression [13–15].

We could show in previous studies, that in melanoma the expression of AGO proteins is reduced compared to healthy tissue or other tumor entities [16]. Especially AGO2, the most abundant AGO protein in human cells, is significantly downregulated in melanoma compared to healthy melanocytes [16, 17]. This has a significant impact on tumorigenic properties, such as migration, and influences the effectiveness of siRNA-mediated regulation in melanoma cells [17]. The molecular mechanisms behind this AGO2 downregulation in melanoma are currently not understood.

Focusing on AGO2 in melanoma cells, we identified a new splice variant of AGO2. This variant, AGO2-ex1/3, lacks exon 2 resulting in the expression of an N-terminally truncated AGO2 protein. Molecular dynamics simulations of the AGO2-ex1/3 protein structure revealed that the truncation of the N-terminus leads to an increased interdomain flexibility which provides new functional options of AGO2-ex1/3 for the miRNA pathway. Our data show that expression of this variant is elevated in melanoma cell lines and that it is associated with melanoma cell viability. Further, AGO2-ex1/3 has a strong impact on a large variety of miRNA target genes implicating a role of this AGO2 variant for miRNA function. This discovery represents an interesting insight into a so far unknown molecular mechanism regarding the miRNA pathway and makes AGO2-ex1/3 an interesting new target for possible future therapeutic approaches in melanoma.

## Materials and methods

### Cell lines, primary cells and tissue samples

The human melanoma cell lines Mel Im (RRID:CVCL_3980), Mel Wei (RRID:CVCL_3981) and HTZ19 (RRID:CVCL_IS45) were cultured in “Dulbecco’s Modified Eagle’s Medium—low glucose” (Sigma-Aldrich Chemie GmbH, Steinheim, Germany) and Mel Juso (RRID:CVCL_1403) in “RPMI-1640” medium (Sigma-Aldrich Chemie GmbH, Steinheim, Germany) at 37 °C and 8% CO_2_. Media were supplemented with 100 U/ml penicillin, 0.1 mg/ml streptomycin and 10% fetal bovine serum (Sigma-Aldrich Chemie GmbH, Steinheim, Germany). RPMI medium was additionally supplemented with 0.2% sodium bicarbonate solution. Melanoma cell lines were regularly tested for mycoplasma contamination.

NHEM were obtained from Lonza Group AG (Basel, Switzerland) and were grown in “MBM™-4 basal medium” (CC-3250) supplied with “MGM™-4 SingleQuots™” supplements (CC-4435) (Lonza Group AG, Basel, Switzerland) at 37 °C and 5% CO_2_ to passage 7 or 8.

Melanoma tissue samples were derived from primary tumors or metastases from three different patients, respectively, provided by the tissue bank of the Institute of Pathology of the University Hospital Regensburg (Regensburg, Germany). Sampling and handling of patient material was carried out according to the ethical principles of the Declaration of Helsinki. The use of human tissue material derived from melanoma patients had been approved by the ethics committee of the University of Regensburg (application numbers 09/11 and 03/151) [16, 17].

### Statistical analyses

Statistical significance was calculated using “GraphPad Prism” (version 5.0.4.533, GraphPad Software Inc., San Diego, USA). For comparison of two groups, in which each pair of values could be unambiguously assigned to a single experiment, a paired *t*-test was performed. For the comparison of two groups, in which the assignment of the pairings was not given due to the experimental conditions, an unpaired *t*-test was applied. For comparison of three or more groups, “One-way analysis of variance (ANOVA)” and subsequent “Tukey’s Multiple Comparison Test” was performed. For analysis of values which were normalized to a control group (set as = 1), one sample *t*-test was used. The number of biological replicates (*n*) used for calculation of statistical significance is indicated in the figure legends respectively.

### Transfection of cells with siRNA or plasmid

For transfection of melanoma cells with plasmids, the “Lipofectamin LTX-Transfection Kit” (Invitrogen, Thermo Fischer Scientific, Waltham, MA, USA) was used with 2.5 µl of “Lipofectamine LTX”, 3 μl “Lipofectamine Plus” and 1 μg of the respective plasmid in 40 µl transfection medium (Dulbecco’s Modified Eagle’s medium without phenol red (PAN-Biotech GmbH, Aidenbach, Germany) supplemented with 1% penicillin/streptomycin) for 220,000–280,000 cells per six-well in 2 ml of the respective cell culture medium. Cells were grown over night before and harvested 24 h after transfection.

For siRNA-transfection the “Lipofectamine RNAiMAX Transfection Reagent” (Invitrogen, Thermo Fischer Scientific, Waltham, MA, USA) was used with 5 μl siRNA (corresponds to 100 pmol siRNA per 6-well) and 5 μl “Lipofectamine RNAiMAX” in 500 µl transfection medium for 120,000–360,000 cells per sixwell in 1.5-ml culture medium. Transfection was performed directly after seeding and cells were harvested after 24–72 h. For re-transfection, cells were counted after 72 h, seeded again in six-wells and transfected with the respective siRNA. The sequences of the siAGO2-ex1/3 (AGCCGGCCCCGGGAAAUCGUG[dT][dT]) and siAGO2-ex1/3_2 (CGGCCCCGGGAAAUCGUGGAA[dT][dT]) were designed to minimize binding to possible other non-specific targets, which was verified by sequence alignment. It was produced by Sigma-Aldrich Chemie GmbH (Steinheim, Germany). As siCtrl, the “AllStars Negative Control siRNA” (QIAGEN GmbH, Hilden, Germany) was used.

Co-transfection of plasmid and siRNA was performed with the “Lipofectamine 2000” Kit (Invitrogen, Thermo Fischer Scientific, Waltham, MA, USA) using 5 μl siRNA, 1 μg of the respective plasmid and 5 μl “Lipofectamine 2000” in 500 µl transfection medium for 220,000–280,000 cells per six-well in 1.5-ml culture medium. Cells were grown over night before transfection and harvested after 24 h.

Additional descriptions of materials and methods can be found in Supplementary File 3.

## Results

### Characterization of a so far unknown splice event in the AGO2 mRNA in melanoma cell lines and patient-derived RNA samples

**Fig. 1:**
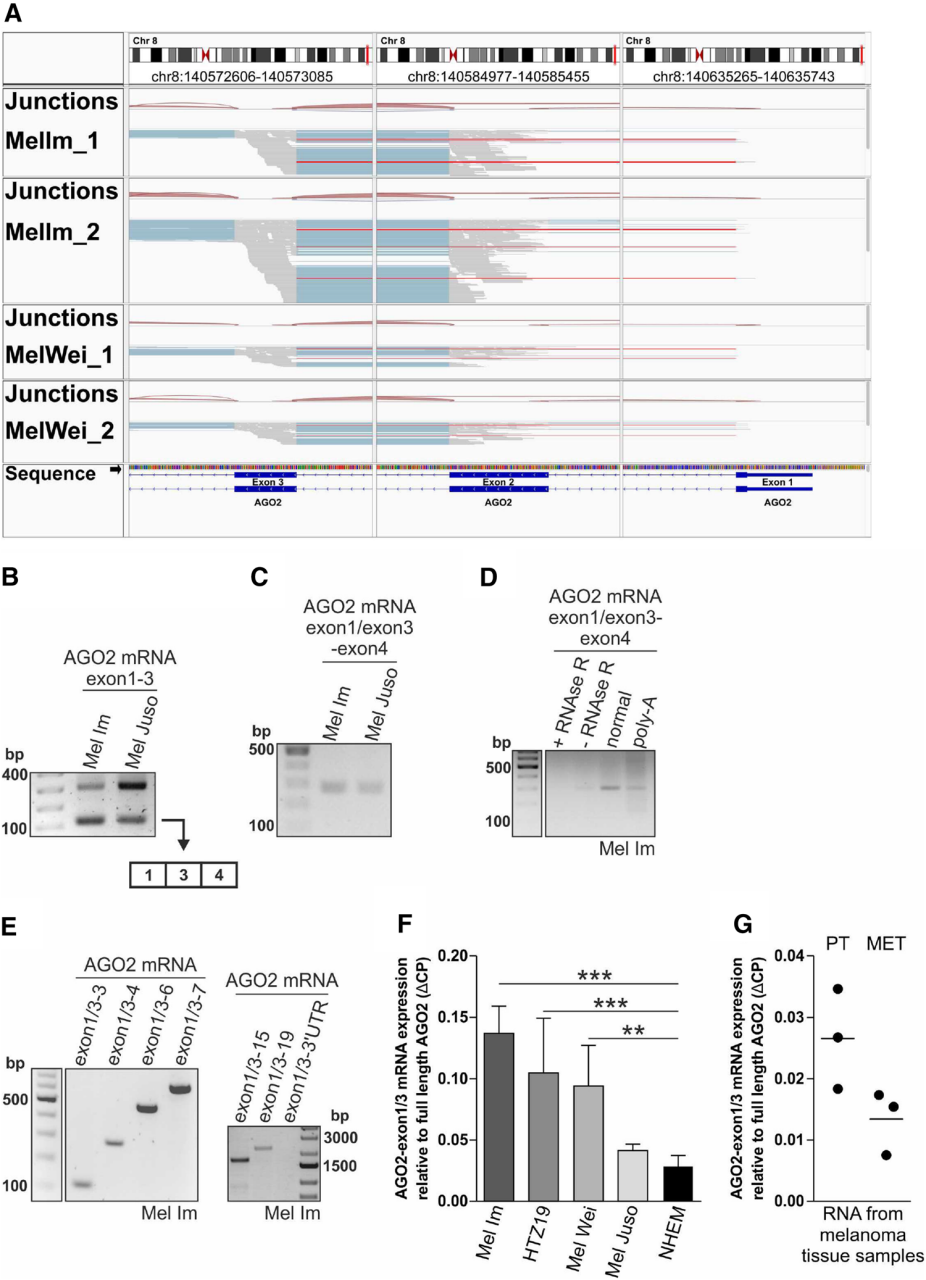
AGO2-ex1/3, a mature, linear AGO2 mRNA variant is expressed in melanoma cells and tissue samples. **A** RNA-Seq reads aligned to the exons1-3 of the AGO2 gene (according to the sequence diagram at the bottom) and the corresponding splice junction track (junctions indicated by red brackets) for two samples of the melanoma cell lines Mel Im (MelIm_1, MelIm_2) and Mel Wei (MelWei_1, MelWei_2), respectively. Reads in exons are depicted as grey lines, gaps in reads caused by splicing out introns are shown as blue lines. Red lines mark reads starting in exon1 and being continued in exon 3, indicating exclusion of exon2. **B** PCR amplification of exon 1-3 of the AGO2 mRNA in the melanoma cell lines Mel Im and Mel Juso. Sequencing of the shorter, 110 bp product revealed an AGO2 variant missing exon2. **C** PCR amplification of the AGO2 variant using a specific forward primer covering the transition between exon1 and exon3 and a reverse primer in exon4. RNA was digested with DNAse I during preparation to exclude contamination with genomic DNA. **D** PCR products of AGO2-ex1/3 after amplification of RNA treated with or without RNase R, normally transcribed RNA and RNA transcribed with a poly(A)-specific primer in the melanoma cell line Mel Im. **E** PCR products of the indicated AGO2 mRNA segments in the melanoma cell line Mel Im show inclusion of all exons except of exon2 in the AGO2-ex1/3 mRNA variant. **F** Relative AGO2-ex1/3 mRNA expression to full-length AGO2 mRNA (ΔCP) analyzed via qRT-PCR in the melanoma cell lines Mel Im, HTZ19, Mel Wei and Mel Juso (mean ± SD,* n* = 3 (Mel Im, Mel Juso), *n* = 5 (HTZ19), *n* = 7 (Mel Wei)) as well as in NHEM samples from eight independent donors (mean ± SD) (**p < 0.01, ***p < 0.001, One-way ANOVA and sub-sequent Tukey’s Multiple Comparison Test). **G** Relative AGO2-ex1/3 mRNA expression to full-length AGO2 mRNA (ΔCP) analyzed via qRT-PCR in 3 primary tumor (PT) and 3 metastasis (MET) derived tissue samples from six independent patients. Mean is shown as line.

The analysis of RNA-sequencing (RNA-seq) data of the AGO2 gene in melanoma cell lines surprisingly revealed reads containing exon1 and exon3 but missing exon2 (Fig. 1A, marked in red). The identified reads indicate an unexpected alternative splicing event in the coding sequence (CDS) of AGO2. Analysis of melanoma patient data from “The Cancer Genome Atlas (TGCA) SpliceSeq Database” (https://bioinformatics.mdanderson.org/TCGASpliceSeq/singlegene.jsp, [18]) also revealed that in some patients (11%) a splicing event occurs in which exon2 is skipped in the AGO2 CDS.

A comparable exon skipping event in the AGO2 gene can only be found in one other exon in the 3´ region of the CDS, where the existence of a shorter AGO2 mRNA variant is already known (https://www.ncbi.nlm.nih.gov/nuccore/NM_001164623.3).

To validate the existence of this so far undescribed splice event of exon 2, PCR amplification of the AGO2 mRNA focusing on the region between exon1 and exon3 was performed in two melanoma cell lines (Fig. 1B). DNA sequencing of the shorter product of 110 bp supported the RNA-Seq results and confirmed the occurrence of an alternative splice event excluding exon2 of the AGO2 mRNA (Fig. 1B). To further confirm the expression of this AGO2 variant, a specific PCR primer was used covering the junction between exon1 and exon3. Again, the AGO2 variant missing exon2, which we named AGO2-ex1/3 in the following, could be amplified with the specific primer in the two melanoma cell lines (Fig. 1C).

To analyze whether AGO2-ex1/3 is a mature, linear mRNA, total RNA was treated with the RNA exonuclease RNase R, which specifically digests only linear RNA and leaves circular RNA. In addition, total RNA was reverse transcribed using a poly(A)-specific primer and subsequently digested with RNase A to eliminate all non-mature mRNAs or other RNA species not containing a poly(A) tail. The PCR amplification of AGO2-ex1/3 with specific primers results in a product using the dN6-transcribed or the poly(A)-specific cDNA, but no product appears in the RNase R treated sample (Fig. 1D). As positive control, we used the HIPK3 gene where the existence of a linear as well as of a circular mRNA version is known [19] (Supplementary Fig. S1). These results indicate that AGO2-ex1/3 is supposed to be a mature, linear mRNA, which is expressed in melanoma cells.

To determine whether AGO2-ex1/3 contains all other exons of the AGO2 mRNA except exon2, the amplification of AGO2-ex1/3 using a specific forward primer on the transition between exon1 and exon3 was extended to the entire AGO2 mRNA length (Fig. 1E). The generated product for each PCR from exon1/3–3 to exon1/3–19 indicates that AGO2-ex1/3 contains, except of exon2, the complete coding sequence of AGO2. We observed no product for the amplification from exon1/3 to the 3´-UTR indicating that the AGO2-ex1/3 variant may differ from AGO2 in its 3-UTR sequence.

Quantitative real-time PCR revealed expression of AGO2-ex1/3 in several different melanoma cell lines and an elevated expression in some cell lines compared to healthy melanocytes (Fig. 1F). Further, expression of AGO2-ex1/3 could also be detected in RNA originating from different melanoma tissue samples (Fig. 1G). These data indicate that AGO2-ex1/3 might play a role for melanoma tumorigenesis.

### AGO2-ex1/3 is translated into an N-terminally truncated AGO2 protein version

**Fig. 2:**
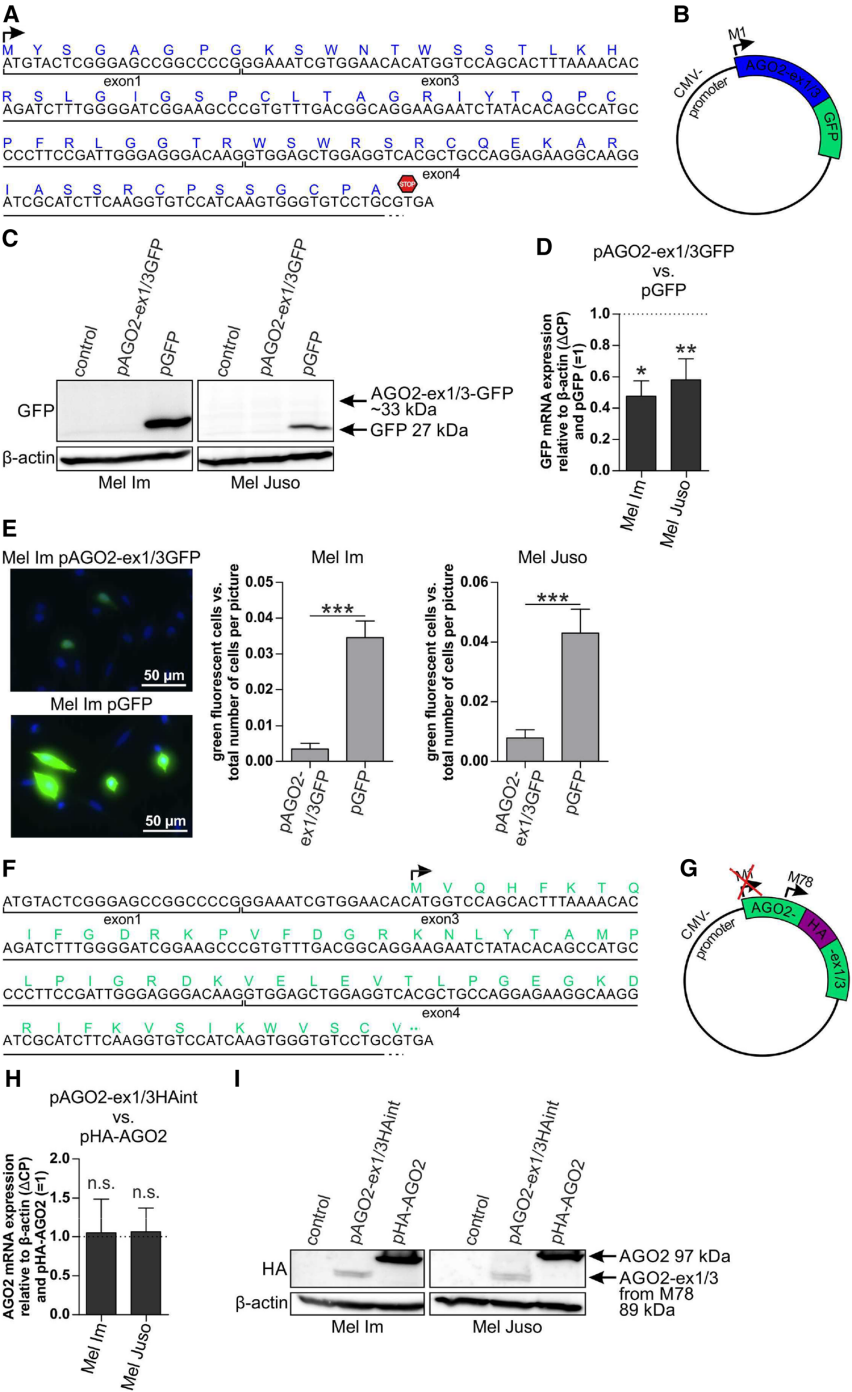
Translation of AGO2-ex1/3 starts from an internal methionine leading to an N-terminally shortened AGO2 protein. **A** MRNA sequence of AGO2-ex1/3 (derived from NM_012154.5) and the respective amino acid sequence (blue) resulting from the first reading frame. **B** Expression plasmid for AGO2-ex1/3 with a c-terminal GFP tag in frame to the first ATG (pAGO2-ex1/3GFP). **C** Western blot of Mel Im and Mel Juso transfected for 24 h with an empty control plasmid, pAGO2-ex1/3GFP or pGFP, stained with antibodies against GFP and β-actin (loading control). **D** GFP mRNA expression in the melanoma cell lines Mel Im and Mel Juso after 24 h transfection with pAGO2-ex1/3GFP relative to β-actin (ΔCP) and to pGFP-transfected cells (= 1), analyzed via qRT-PCR (mean ± SD from *n* = 3 (Mel Im) or *n* = 4 (Mel Juso), **p < 0.01, one sample t-test compared to 1). **E** Immunofluorescence imaging of pAGO2-ex1/3GFP- or pGFP-transfected cells (green), cell nuclei were stained with DAPI (blue). The evaluation shows the average number of fluorescent cells per picture (mean ± SEM from *n* = 4 with 8–10 pictures, respectively, ***p < 0.001, unpaired *t*-test). **F** MRNA sequence of AGO2-ex1/3 and the respective amino acid sequence (green) resulting from a second reading frame starting from ATG (39) which refers to M78 of AGO2. **G** Expression plasmid for AGO2-ex1/3 with an internal HA-tag, which is only in frame if translation starts from M78 (pAGO2-ex1/3HAint). **H** AGO2 mRNA expression in the melanoma cell lines Mel Im and Mel Juso after 24 h transfection with pAGO2-ex1/3HAint relative to β-actin (ΔCP) and to pHA-AGO2-transfected cells (= 1), analyzed via qRT-PCR (mean ± SD, *n* = 5, *n.s.* not significant, one sample t-test compared to 1). **I** Western blot of Mel Im and Mel Juso transfected with an empty control plasmid, pAGO2-ex1/3HAint or pHA-AGO2 for 24 h stained with antibodies against HA and β-actin (loading control)

To analyze the functional relevance of AGO2-ex1/3 expression on melanoma cells, we first investigated in which way the altered AGO2-ex1/3 mRNA could be translated. AGO2-ex1/3 contains the same first methionine (M) as the full-length AGO2 mRNA but due to a frameshift after exon1 the translation stops after only 73 amino acids (Fig. 2A). To examine translation of this peptide, we generated an expression plasmid carrying the AGO2-ex1/3 sequence from the first AUG until the first in-frame stop codon and replaced the stop codon by an in-frame C-terminal GFP-tag (Fig. 2B). After transfection of this construct into the melanoma cell lines Mel Im and Mel Juso, the AGO2-ex1/3-GFP fusion protein should be detected in the Western blot at a size of about 33 kDa. Interestingly, no signal could be identified at the expected height (Fig. 2C), whereas a construct with only GFP at the size of 27 kDa is well expressed. On mRNA level, GFP expression is reduced to about 50% after pAGO2ex1/3GFP transfection compared to pGFP transfection (Fig. 2D). Fluorescence microscope images of the transfected cells show only a very faint GFP signal in pAGO2-ex1/3GFP-transfected cells compared to pGFP and a significantly lower number of GFP positive cells at all (Fig. 2E) also demonstrating a very weak expression of the AGO2-ex1/3-GFP fusion protein. These data indicate that the first M of AGO2-ex1/3 seems to be weakly used for translation.

In addition to M1, the AGO2-ex1/3 mRNA contains a second possible start codon at position 39 which refers to M78 in the full-length AGO2 mRNA (Fig. 2F). This M78 also exhibits a Kozak sequence, which makes translation start at this position probable [20]. Further, Mourelatos et al. could identify an AGO2-like protein via mass spectrometry co-precipitating with Gemin3 in HeLa cells that starts with M78 [21]. In the expressed sequence tag (EST) database, an AGO2 mRNA sequence can be found, identical to our AGO2-ex1/3 sequence, where translation prediction results in a protein version using this M78 as translational start site (https://www.ncbi.nlm.nih.gov/nuccore/XM_017013317.2?report=genbank). To find out, whether AGO2-ex1/3 can be translated from this second start codon referring to M78 in melanoma cells, we generated an expression plasmid with the complete AGO2-ex1/3 mRNA sequence containing an additional internal HA sequence in exon6 (pAGO2-ex1/3HAint, Fig. 2G). The HA-sequence is only in frame when AGO2-ex1/3 is translated from M78. The HA-tag was placed into a region of the known AGO2 structure that forms an outward facing loop to affect protein folding of the putative AGO2-ex1/3 protein as little as possible.

We transfected pAGO2-ex1/3HAint into the melanoma cell lines Mel Im and Mel Juso. As control, we transfected the normal AGO2 sequence bearing an N-terminal HA-tag (pHA-AGO2) with a known protein size of 97 kDa.

On mRNA level, a comparable AGO2 mRNA expression can be observed for cells transfected with pAGO2-ex1/3HAint relative to pHA-AGO2-transfected cells (Fig. 2H). On protein level, after pAGO2-ex1/3HAint transfection a signal in the Western blot referring to a protein size of about 89 kDa can be detected (Fig. 2I). The observed band size of 89 kDa after pAGO2-ex1/3HAint-transfection fits to an HA-tagged AGO2-ex1/3 protein starting at M78. It indicates that during translation of AGO2-ex1/3 the first M can be skipped. These data imply that M78 may be the commonly used translational start site of AGO2-ex1/3 in melanoma cells.

**Fig. 3:**
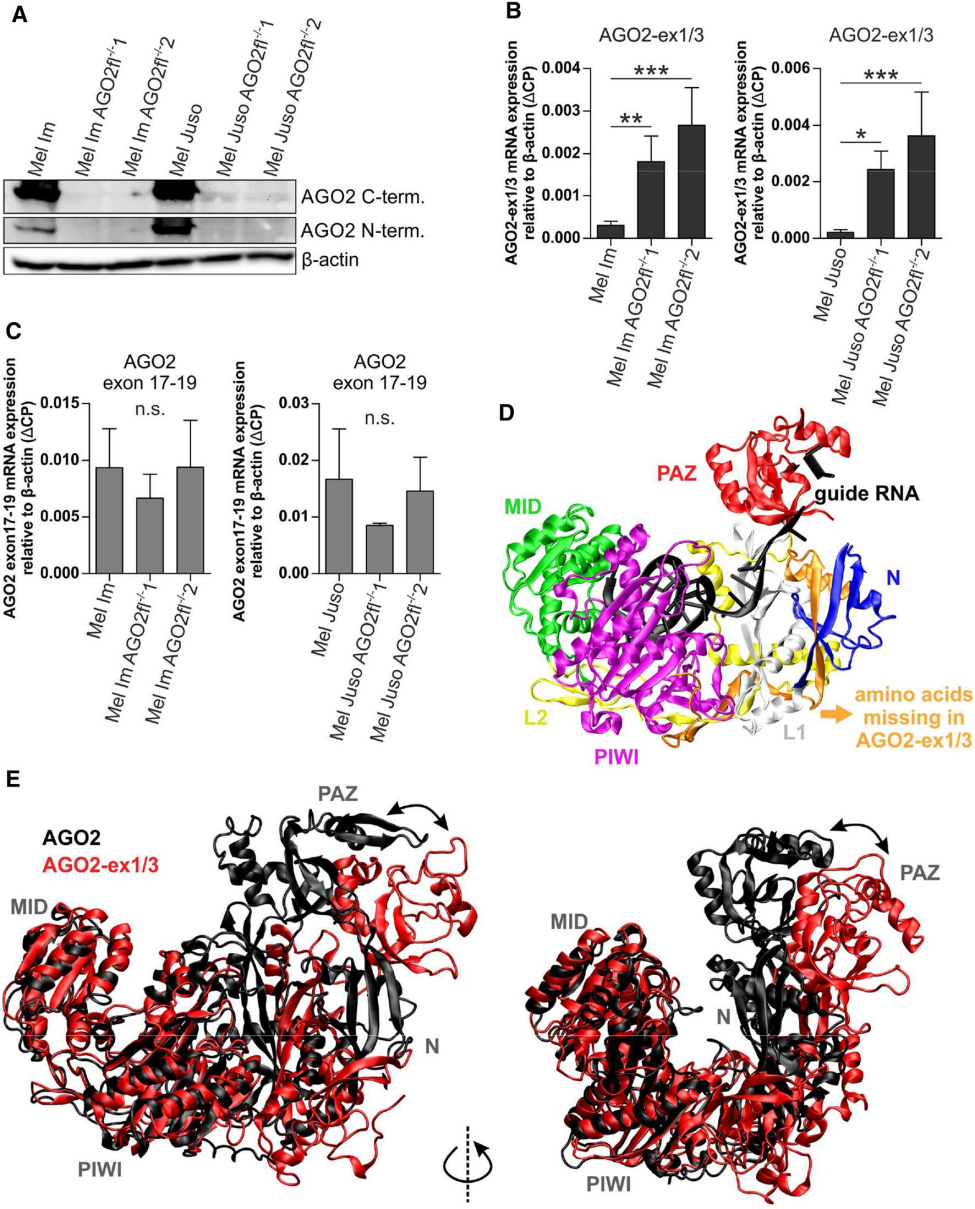
AGO2-ex1/3 protein expression, structure and dynamics. **A** Western blot showing AGO2 protein expression in wild-type melanoma cell lines and AGO2-ex1/3 expression in the indicated full-length AGO2 knockout (AGO2fl^−/−^) cell lines using antibodies against a C-terminal AGO2 epitope, an N-terminal AGO2 epitope and β-actin (loading control). **B** MRNA expression of AGO2-ex1/3 and **C** a 3´-region of the AGO2 mRNA in the indicated AGO2 full-length knockout or wildtype melanoma cell lines analyzed via qRT-PCR relative to β-actin (ΔCP) (mean ± SD, *n* = 5–6 (Mel Im + AGO2 knockout cell lines), *n* = 3–6 (Mel Juso + AGO2 knockout cell lines), *p < 0.05, **p < 0.01, ***p < 0.001 and *n.s.* not significant, One-way ANOVA and subsequent Tukey’s Multiple Comparison Test). **D** Protein structure of AGO2 bound to a guide RNA (PDB-ID: 4z4d; [22]). Protein domains are depicted in different colors: N-domain (blue), L1 linker (white), PAZ domain (red), L2 linker (yellow) MID domain (green), Piwi domain (violet). The N-terminal residues 1–77 missing in the truncated form AGO2-ex1/3 are colored orange. RNA-residues resolved in the structure are depicted in black. **E** Superimposition of the MID and PIWI domain in the final structures of the Gaussian-accelerated molecular dynamics simulation: full-length AGO2 (black), AGO2-ex1/3 (red). Two views of the same overlay are shown. The different overall protein conformation is indicated by arrows

To find out, if the N-terminally truncated AGO2 protein version is also endogenously translated from the AGO2-ex1/3 mRNA in melanoma cells, we used an antibody targeting a C-terminal region of the AGO2 protein. As the difference in protein size between endogenous AGO2 and AGO2-ex1/3 translated from M78 is only very small (~ 7 kDa), both proteins could hardly be distinguished via Western blot (Fig. 3A, lane 1 and 4). To be able to analyze endogenous AGO2-ex1/3 protein expression in melanoma cells, we performed a genetic knockout of full-length AGO2 in the melanoma cell lines Mel Im and Mel Juso using CRISPR/Cas9. The knockout affects the region of exon2 of the AGO2 gene, resulting in a loss of full-length (fl) AGO2, but the splice variant AGO2-ex1/3, missing exon 2, can still be formed (Supplementary Fig. S2A). A successful homozygous deletion was verified via DNA sequencing (Supplementary Fig. S2B) and PCR using genomic DNA (Supplementary Figure S2C) in two clones of each knockout cell line respectively (in the following called: Mel Im AGO2fl^−/−^ 1 and 2 and Mel Juso AGO2fl^−/−^ 1 and 2). On mRNA level no expression of full-length AGO2 mRNA containing exon 2 can be detected in the knockout cell lines (Supplementary Figure S2D). PCR analysis amplifying exon 1–3 of AGO2 shows that the AGO2-ex1/3-version (110 bp) is still expressed in the AGO2fl^−/−^ melanoma cell lines but the longer PCR product containing exon1, 2 and 3 (303 bp) disappeared (Supplementary Figure S2E).

On protein level, full-length AGO2 expression, examined with an antibody against the N-terminal region of AGO2 cannot be observed anymore (Fig. 3A). With the C-terminal AGO2 antibody a band could be detected, especially in the Mel Juso knockout cell lines Mel Juso AGO2fl^−/−^ 1 and 2, fitting to the 7 kDa smaller AGO2-ex1/3 protein variant starting from M78 (Fig. 3A). This indicates endogenous protein expression of AGO2-ex1/3 starting from the second M.

Via qRT-PCR analysis we observed a significant increase in AGO2-ex1/3 mRNA expression after the AGO2 full-length knockout (Fig. 3B). Further, a similar expression level of the 3’-end part of the AGO2 mRNA could be detected in the AGO2 knockout cells compared to the wildtype cells (Fig. 3C). These data indicate that the knockout of full-length AGO2 does not reduce the total amount of the AGO2 transcript. It appears that the expression of AGO2-ex1/3 on mRNA level compensates for the loss of AGO2. It can be concluded that in these cells the AGO2 gene is, due to the genomic lack of exon 2, predominantly spliced into the AGO2-ex1/3 variant.

### The truncation of N-terminal residues leads to an increased interdomain flexibility in the AGO2-ex1/3 protein structure

In the following, we wanted to assess to which extend the truncation of the first 77 amino acids impacts AGO2-ex1/3 protein structure. First information about the structural effect of the 77-residue truncation was gained from a visual inspection of the existing AGO2 crystal structure (Fig. 3D, [22]). The truncation includes parts of the N-domain (residues 53–138) as well as the entire N-terminal segment (residues 1–52), which tightly interacts with the L2-linker (residues 348–444). The loss of residues 53–77 belonging to the N-domain affects a region, which forms contacts to the guide strand RNA in full-length AGO2. The analysis of the static structure suggests a direct effect of the truncation on structure and function of the N-domain. To gain information on potential remote effects of the truncation on the structure and dynamics of the remaining domains, we performed molecular dynamics simulations.

The analysis revealed that the structure of the PAZ-, MID- and PIWI-domain remained mainly intact in AGO2-ex1/3 (Fig. 3E). However, an enhanced interdomain flexibility was observed, which can mainly be attributed to structural fluctuations of the L1 and L2 linker regions (Fig. 3E and supplementary file 2). Thus, the truncation enhances the flexibility in the cleft between the N + PAZ- and the MID + PIWI-lobe in AGO2-ex1/3.

### AGO2-ex1/3 influences melanoma cell growth

**Fig. 4:**
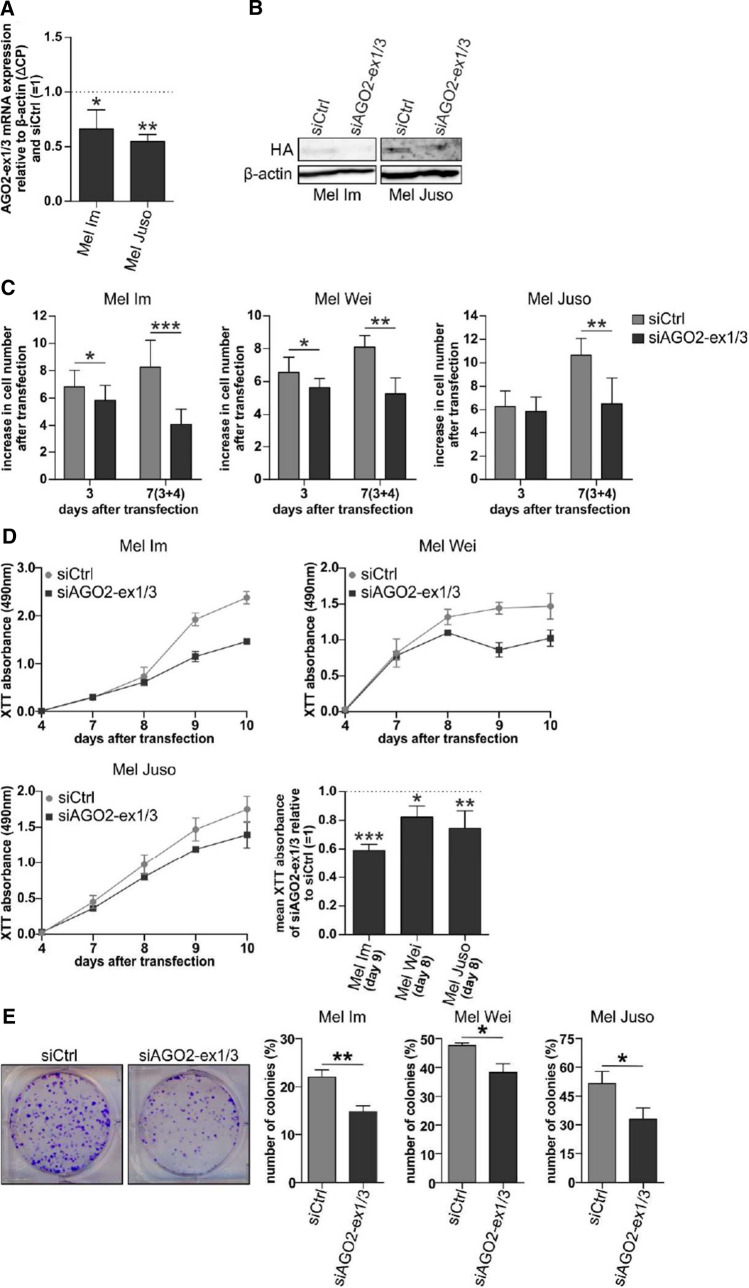
A siRNA-mediated knockdown of the AGO2-ex1/3 variant impairs melanoma cell growth. **A** Relative AGO2-ex1/3 mRNA expression to β-actin (ΔCP) and siCtrl (= 1), analyzed via qRT-PCR in the melanoma cell lines Mel Im and Mel Juso after 24 h transfection with siAGO2-ex1/3 (mean ± SD, *n* = 4 (Mel Im), *n* = 3 (Mel Juso), *p < 0.05, **p < 0.01, one sample t-test compared to 1). **B** Western blot stained with antibodies against HA or β-actin (loading control) of Mel Im and Mel Juso transfected with pAGO2-ex1/3HAint and siAGO2-ex1/3 or a control siRNA for 24 h. **C** Cell number of indicated melanoma cell lines relative to seeded cell number after 3 or 7 days (with re-transfection at day 3) of transfection with siAGO2-ex1/3 or a control siRNA (mean ± SD, *n* = 20 and 9 (Mel Im), *n* = 8 and 4 (Mel Wei), *n* = 10 and 5 (Mel Juso), *p < 0.05, **p < 0.01, ***p < 0.001, unpaired *t*-test). **D** XTT viability assay of indicated melanoma cell lines showing XTT absorbance at displayed time points after transfection with siAGO2-ex1/3 or a control siRNA (with re-transfection at day 3). Graphs show one exemplarily assay for each cell line with three technical replicates. Bars show a summary of absorbance values of siAGO2-ex1/3-treatment relative to siCtrl at day 8 or 9 (mean ± SD, *n* = 4 (Mel Im) or *n* = 5 (Mel Juso, Mel Wei), *p < 0.05, **p < 0.01 and ***p < 0.001, one sample *t*-test compared to 1). **E** Clonogenic assay showing the number of colonies relative to the number of seeded cells (%) of indicated melanoma cell lines after 7 days. Pictures represent an exemplarily staining of colonies of Mel Im. The cells were transfected with siAGO2-ex1/3 or siCtrl and re-transfected at day 3 before starting the assay at day 4 (mean ± SD, *n* = 7 (Mel Im), *n* = 4 (Mel Wei) or *n* = 5 (Mel Juso) with two pictures each, *p < 0.05, **p < 0.01, unpaired *t*-test)

To determine which role AGO2-ex1/3 expression plays in melanoma cells, we designed an siRNA (siAGO2-ex1/3) that specifically targets the mRNA of the AGO2-ex1/3 splice variant at the junction between exon1 and exon3. Transfection of the melanoma cell lines Mel Im and Mel Juso with siAGO2-ex1/3 leads to a reduction of AGO2-ex1/3 mRNA expression of about 34–45% after 24 h (Fig. 4A). Also, on protein level siAGO2-ex1/3 reduces expression from pAGO2-ex1/3HAint (Fig. 4B). The expression of full-length AGO2 is not diminished after siAGO2-ex1/3 transfection on mRNA (Supplementary Figure S3A) or on protein level (Supplementary Figure S3B).

Transfection of the melanoma cell line Mel Im with the siAGO2-ex1/3 resulted in a significantly decreased cell number after a transfection period of about 72 h (Fig. 4C). This effect could also be observed in other melanoma cell lines (Mel Juso and Mel Wei) and was even increased after a long time of AGO2-ex1/3 downregulation of 7 days. This observation indicates that AGO2-ex1/3 is important for melanoma cell growth. In addition, starting from about 3 days after transfection, it was noticed that the cells changed their morphology, which was enhanced over a longer transfection period of 7 days (Supplementary Fig. S4A). A staining of filamentous actin showed a significantly increased cell area after siAGO2-ex1/3 transfection, as well as the formation of stress fiber-like structures in the cytoplasm (Supplementary Fig. S4B, C).

To analyze the effect on growth in melanoma cells after AGO2-ex1/3 knockdown in more detail, we measured cell viability using an XTT-assay. For all three investigated melanoma cell lines a significant reduction of XTT absorbance could be observed 8–9 days after siAGO2-ex1/3 transfection compared to siCtrl-treated cells (Fig. 4D). This hints to a lower viability or proliferation of melanoma cells with reduced AGO2-ex1/3 expression.

To further investigate this effect on growth, siAGO2-ex1/3-transfected cells were analyzed using real-time cell analysis (RTCA). The results show a reduction of cell growth in the melanoma cell lines Mel Im, Mel Wei and Mel Juso (Supplementary Figure S5A-C) and confirm our previous observations.

An important property during the growth of tumor cells is the ability to divide starting from a single cell. This ability was measured in a clonogenic assay, where siAGO2-ex1/3-transfected melanoma cells show a significantly reduced colony number (Fig. 4E). These data again support the occurrence of a growth defect of melanoma cells missing AGO2-ex1/3 expression.

To confirm the effect a knockdown of AGO2-ex1/3 has on melanoma cell growth and to rule out off-target effects, we designed a second siRNA targeting AGO2-ex1/3 which is shifted by a few nucleotides from the siAGO2-ex1/3. Transfection of this siAGO2-ex1/3_2 into the melanoma cell lines Mel Im and Mel Wei resulted in reduced cell proliferation analyzed by XTT viability assay (Supplementary Fig. S6A–C). These data confirm that a decrease in cell proliferation is specifically induced by diminished expression of the AGO2 splice variant AGO2-ex1/3 in melanoma cells.

### A knockdown of AGO2-ex1/3 in melanoma cells induces cell death

**Fig. 5:**
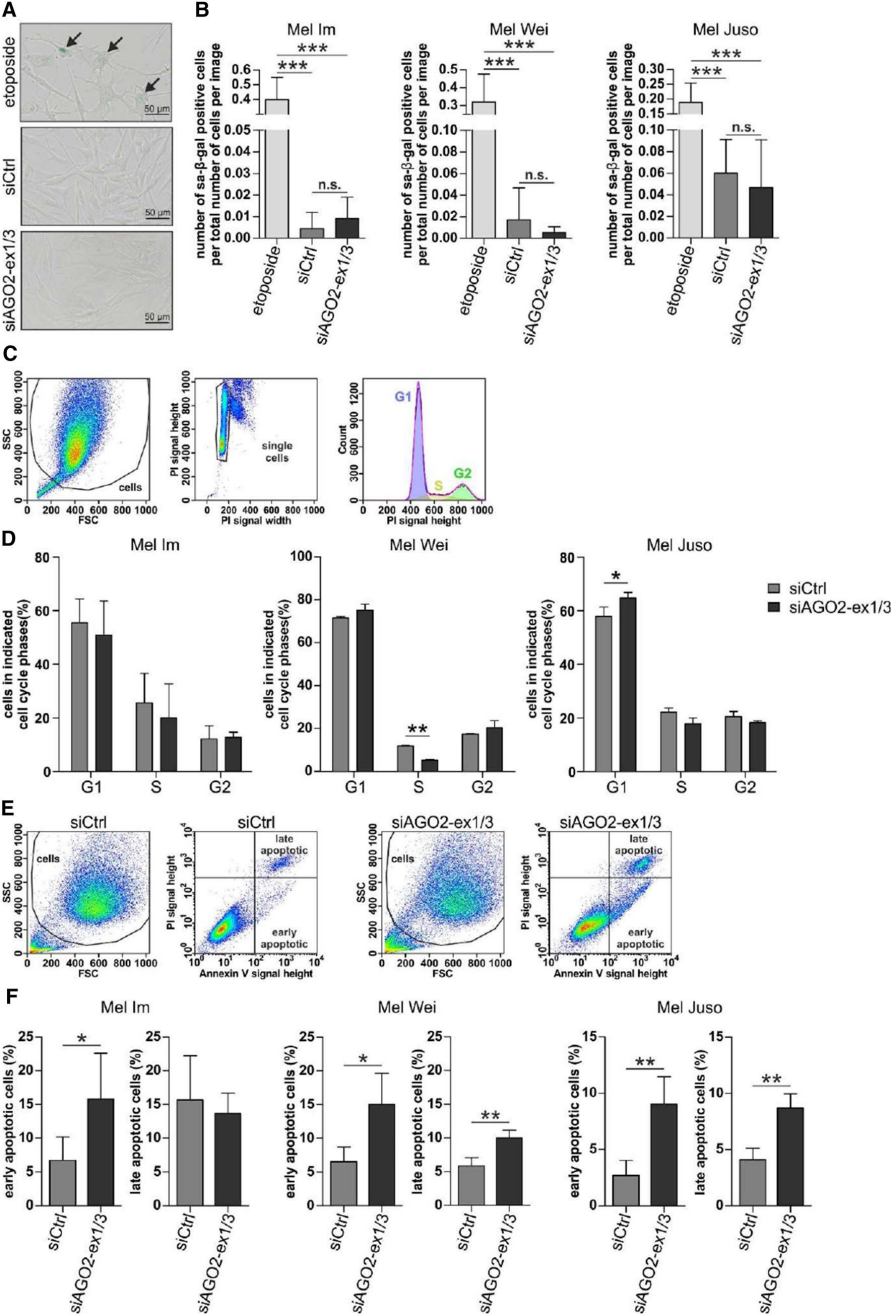
Knockdown of AGO2-ex1/3 does not induce cell cycle arrest or senescence but induces apoptosis. **A** Senescence-associated β-galactosidase (sa-β-gal) positive, blue stained cells indicated by arrows, exemplarily shown for Mel Wei after 48-h treatment with etoposide or 72 h siRNA transfection. **B** Average number of sa-β-gal positive cells relative to total number of cells per image. Cells were treated for 48 h with etoposide or transfected for 3 days (Mel Im 3, 3 + 4 or 3 + 4 + 3 days) with siAGO2-ex1/3 or a control siRNA (mean ± SD, *n* = 3 with 4–7 pictures each, ***p < 0.001, *n.s.* not significant, One-way ANOVA and subsequent Tukey’s Multiple Comparison Test). **C** Cell cycle analyzed with propidium iodide (PI) staining of the DNA content and flow cytometry. The pictures show exemplarily in Mel Wei (siCtrl) the gating of cells in the forward (FSC) and sideward (SSC) scatter, the gating of single cells and the cell cycle analysis using the Dean–Jett–Fox model. **D** Average cell number allocated to the respective cell cycle phases (%) determined as depicted in C of the indicated melanoma cell lines transfected for 7 days (with re-transfection at day 3) with siAGO2-ex1/3 or a control siRNA (mean ± SD, *n* = 3 (Mel Juso, Mel Wei) or *n* = 4 (Mel Im), *p < 0.05, **p < 0.01, One-way ANOVA and subsequent Tukey’s Multiple Comparison Test). **E** Analysis of early and late apoptotic cells via propidium iodide (PI) and annexin V-FITC staining of living cells and flow cytometry, exemplarily shown for Mel Juso. **F** Number of early and late apoptotic cells (%) determined as depicted in (**E**) of the indicated melanoma cell lines after 7 days of transfection (with re-transfection after 3 days) with siAGO2-ex1/3 or a control siRNA (mean ± SD, *n* = 4 (Mel Im), *n* = 3 (Mel Wei), *n* = 5 (Mel Juso), *p* < 0.05, **p < 0.01, paired *t*-test)

One possible explanation for the reduced cell growth of melanoma cells after downregulation of AGO2-ex1/3 would be the occurrence of a cell cycle arrest, e.g., by induction of senescence. To determine if the cells undergo senescence, siAGO2-ex1/3-transfected cells were examined for senescence-associated β-galactosidase (sa-β-gal) activity. As positive control, cells were treated with etoposide, a topoisomerase inhibitor which induces cellular senescence caused by DNA damage [23]. Staining of sa-β-gal in the siAGO2-ex1/3-transfected melanoma cell lines Mel Im, Mel Wei and Mel Juso shows no increase in senescent cells compared to siCtrl-transfected cells (Fig. 5A, B). In contrast, after treatment with etoposide an accumulation of sa-β-gal positive cells is visible. These data exclude senescence as a cause for the observed effects on growth after AGO2-ex1/3 downregulation.

To determine whether the reduced cell growth is caused by other modifications of the cell cycle, the cellular DNA was stained using propidium iodide (PI) in siAGO2-ex1/3-transfected cells compared to siCtrl and cell cycle phases were determined on the basis of the DNA content using flow cytometry (Fig. 5C). The analysis of cell cycle showed no remarkable increase of cells in G1 or G2 cell cycle phases in Mel Im and Mel Wei indicating no presence of a cell cycle arrest (Fig. 5D). Mel Wei cells show a significant decrease in cells in the S phase which implies fewer proliferating cells. Only in the melanoma cell line Mel Juso a small but significant increase of cells in the G1 phase can be observed after siAGO2-Ex1/3 transfection indicating the occurrence of a cell cycle arrest. In summary, the lack of or the minor effects on cell cycle arrest cannot explain the huge differences in cell growth after AGO2-ex1/3 knockdown that have been observed in the experiments before. However, when analyzing the cell cycle data, an increase in the number of cells with lower DNA content than cells in G1 (SubG1) was observed indicating dead cells (Supplementary Fig. S7A, B).

Consequently, we analyzed the occurrence of apoptosis in melanoma cells after transfection with the siAGO2-ex1/3. Early and late apoptotic cells were identified using annexin V and PI staining of living cells via flow cytometry (Fig. 5E). We observed a significant increase in early apoptotic cells after 7 days of siAGO2-ex1/3 transfection compared to siCtrl in the melanoma cell line Mel Im (Fig. 5F). In the cell lines, Mel Wei and Mel Juso early and late apoptotic cells were significantly elevated after siAGO2-ex1/3 transfection. This observation suggests apoptosis as the main cause of the reduced cell number and viability of melanoma cells missing AGO2-ex1/3 expression.

### AGO2-ex1/3 knockdown has a profound effect on miRNA target genes

To understand the molecular mechanisms affected by knockdown of AGO2-ex1/3, which induce the observed reduction in proliferation and increase in apoptosis, we performed RNA-Seq after siAGO2-ex1/3 transfection in the melanoma cell line Mel Juso.

**Fig. 6:**
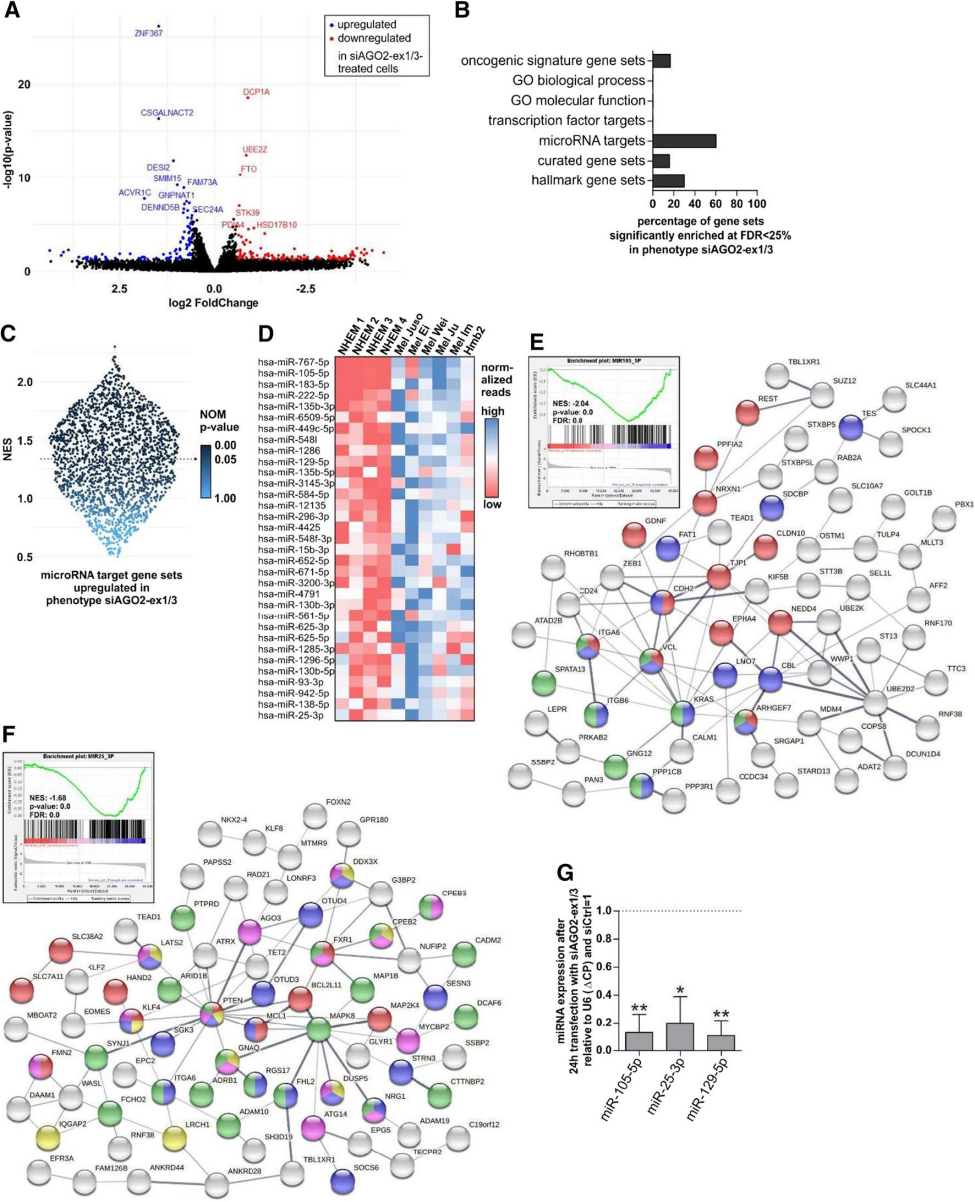
RNA-Seq analysis reveals a significant regulation of miRNA target genes after AGO2-ex1/3 knockdown. **A** Differentially expressed genes in siAGO2-ex1/3-transfected Mel Juso cells compared to siCtrl-transfected cells analyzed by RNA-Seq. Significantly (*p*-value < 0.05) and strongly (> 1.5 fold regulated, corresponds to log2 FoldChange > 0.585 or < − 0.585 respectively) upregulated genes are marked in blue and downregulated genes in red. **B** Gene set collections from the “Molecular Signatures Database” where the associated gene sets are significantly enriched in siAGO2-ex1/3-transfected cells with a false discovery rate (FDR) below 0.25, analyzed via gene set enrichment analysis (GSEA). **C** Violin scatter plot showing the normalized enrichment score (NES) and the nominal (NOM) p value for miRNA target gene sets enriched in siAGO2-ex1/3-transfected cells. Significant gene sets below *p* < 0.05 are indicated by arrow. **D** Heatmap showing normalized read counts of miRNAs significantly upregulated in melanoma cell lines derived from primary tumors (Mel Juso, Mel Ei, Mel Wei) compared to NHEM (mean from four independent samples) whose target genes are simultaneously significantly enriched in siAGO2-ex1/3 transfected Mel Juso cells (according to **C**). **E** Enrichment plot showing the enrichment score for target genes of miR-105-5p. The normalized enrichment score (NES), *p* value and false discovery rate (FDR) are indicated. Red color indicates genes enriched in siCtrl-, blue color depicts genes enriched in siAGO2-ex1/3-transfected cells. STRING protein network analysis of miR-105-5p target genes (derived from the molecular signatures database miR-105-5p gene set) showing core enrichment in siAGO2-ex1/3 transfected Mel Juso cells: Red spheres show genes significantly involved in cell junction organization (GO:0034330), blue spheres genes located at focal adhesions (GO:0005925) and green spheres genes engaged in the regulation of actin cytoskeleton (KEGG pathway: hsa04810). **F** Enrichment plot of miR-25-3p target genes as described in (**E**). STRING protein network analysis of miR-25-3p target genes showing core enrichment after AGO2-ex1/3 knockdown: Red spheres indicate genes significantly involved in the regulation of cellular response to stress (GO:0080135), blue spheres in the negative regulation of signal transduction (GO:0009968), yellow spheres in the negative regulation of catalytic activity (GO:0043,086), pink spheres in the negative regulation of protein metabolic processes (GO:0,051,248) and green spheres genes located at cell junctions (GO:0030054). **G** MiRNA expression after 24-h transfection of Mel Juso with siAGO2-ex1/3 relative to U6 snRNA (ΔCP) and siCtrl (= 1), analyzed via qRT-PCR (mean ± SD, *n* = 3, *p < 0.05, **p < 0.01, one sample *t*-test compared to 1)

Figure 6A shows genes significantly (*p*-value < 0.05) and strongly upregulated (more than 1.5 fold regulated, corresponds to log2 FoldChange > 0.585) in siAGO2-ex1/3- compared to siCtrl-treated cells marked in blue (see also Supplementary File 4 for differential expression analysis). Strongly downregulated (log2 FoldChange < − 0.585) genes are marked in red.

To validate expression of those genes, we performed qRT-PCR for the six most strongly and significantly upregulated genes (ZNF367, CSGALNACT2, DESI2, SMIM15, FAM73A and ACVR1C) after 24 h, 48 h and 72 h transfection with siAGO2-ex1/3 in Mel Juso cells (Supplementary Fig. S8). For all genes, we could confirm an upregulation after loss of AGO2-ex1/3 expression which is even increased after a longer transfection period.

A “STRING” protein network analysis revealed that neither the significantly and strongly up- nor downregulated genes after siAGO2-ex1/3 transfection cluster together in a specific biological network or pathway (Supplementary Fig. S9A, B). To further examine distinct molecular connections between the genes affected by a reduced expression of AGO2-ex1/3, we performed gene set enrichment analysis (GSEA). The analysis of several gene set collections from the “Molecular Signatures Database” (MSigDB) also revealed no significant enrichment of certain biological processes or molecular function, but a strong significant enrichment of miRNA target gene sets (Fig. 6B). Of all 2343 miRNA target gene sets which were included into the analysis, 1407 gene sets (60%) were significantly enriched in siAGO2-ex1/3 treated cells with a false discovery rate (FDR) < 0.25 and none in the siCtrl. The proportion of miRNA target gene sets which are predominantly regulated in siAGO2-ex1/3 and not in the siCtrl are shown in Fig. 6C. Accordingly, AGO2-ex1/3 seems to be substantially involved in microRNA target gene regulation.

The miRNA target gene enrichment after siAGO2-ex1/3 transfection suggests that a loss of AGO2-ex1/3 contributes to downregulation or loss of function of the associated microRNAs. To have the ability to trigger the observed cellular effects, such as decreased proliferation and the occurrence of apoptosis, the downregulation of AGO2-ex1/3 would have to lead to loss of function of those microRNAs significantly involved in melanoma cell growth. To identify such miRNAs, we analyzed a connection of miRNAs which are highly expressed in melanoma cell lines compared to NHEM with a deregulation of microRNA target genes after AGO2-ex1/3 knockdown. Figure 6D shows a heatmap of microRNAs whose target genes are significantly enriched after siAGO2-ex1/3 transfection and which are simultaneously significantly overrepresented in melanoma cells derived from primary tumors compared to healthy NHEM. Among those, miR-105-5p is the miRNA whose target genes are most strongly enriched after AGO2-ex1/3 knockdown (Fig. 6E, NES − 2.04) and which is also significantly upregulated in melanoma cells (Fig. 6D). A STRING protein network analysis of the miR-105-5p target genes which are enriched in siAGO2-ex1/3 treated Mel Juso cells (GSEA core enrichment) revealed genes which were significantly involved in cell junction organization (GO:0034330; Fig. 6E, red spheres), are located at focal adhesions (GO:0005925, Fig. 6E, blue spheres) and are engaged in the regulation of actin cytoskeleton (KEGG pathway: hsa04810, Fig. 6E, green spheres). Therefore, those genes could potentially be responsible for the observed cell phenotype after AGO2-ex1/3 loss regarding the enlarged cell area and stress-fiber-like actin rearrangements.

MiR-25-3p is the most abundant miRNA in melanoma cells (29,562 mean normalized reads) in the list of miRNAs whose target genes are significantly enriched after AGO2-ex1/3 knockdown and which are also significantly upregulated in primary melanoma cells compared to NHEM (Fig. 6D). The target genes of miR-25-3p, which are enriched after AGO2-ex1/3 knockdown, are significantly involved in the regulation of cellular response to stress (GO:0080135, Fig. 6F, red spheres), and in the negative regulation of several biological processes such as signal transduction (GO:0009968, Fig. 6F, blue spheres), catalytic activity (GO:0043086 Fig. 6F, yellow spheres) or protein metabolic processes (GO:0051248, Fig. 6F, pink spheres). An upregulation of those genes due to the loss of function of their regulating miRNA could be playing a considerable role in the reduced growth observed after AGO2-ex1/3 knockdown. Further, several of those genes are also located at cell junctions (GO:0030054, Fig. 6F, green spheres).

To analyze if the observed upregulation of miRNA target genes after knockdown of AGO2-ex1/3 is indeed a direct correlation of loss of function of the respective miRNAs, we examined miRNA expression in Mel Juso cells after siAGO2-ex1/3 transfection. Three selected miRNAs, miR-105-5p, miR-25-3p and miR-129-5p were strongly and significantly downregulated after loss of AGO2-ex1/3 (Fig. 6G) indicating a correlation between loss of the AGO2 splice variant and miRNA expression.

Considering that the most perturbed genes after AGO2-ex1/3 knockdown cannot be assigned to a single common signaling pathway and that many miRNA target genes are mainly affected by the knockdown, it seems likely that AGO2-ex1/3 is involved in the regulation of function of many different miRNAs. This suggests that the observed apoptosis after AGO2-ex1/3 knockdown is not caused by the perturbation of one single cellular pathway, but rather the deregulation of a large variety of miRNAs involved in several different cellular mechanisms, ultimately resulting in proliferation arrest and cell death. Therefore, AGO2-ex1/3 may play a considerable role for general miRNA function in melanoma cells. This makes this newly identified AGO2 splice variant an interesting target gene in melanoma and an important molecule considering small RNA-based therapies.

## Discussion

In human cells, a large proportion of all expressed genes are alternatively spliced and many splice products are specifically associated with tumorigenesis, aggressiveness, and invasiveness [24]. This study identified a new splice variant of the miRNA-binding protein AGO2 in melanoma cells, missing exon 2 in the CDS. Our data revealed that the exclusion of exon 2 in AGO2-ex1/3 leads to a skipping of the first AUG (M1) during translation and usage of a second, downstream start codon (referring to M78 in the full-length AGO2 protein) for the start of protein synthesis. Alternative translation initiation is very common in human transcripts. It can contribute to genetic diversity or may be a cellular response to specific external circumstances, such as stress [25]. The development of deep sequencing techniques in recent years has made it possible to analyze ribosome-bound RNA fragments at single nucleotide resolution, demonstrating that more than half of all human transcripts contain more than one translation initiation site [25]. The usage of a second, downstream open reading frame (ORF), as it is the case for AGO2-ex1/3, can occur when the first AUG is only weak and is bypassed by the ribosome scanning the mRNA for translation [26]. Since the first and the second AUG in AGO2-ex1/3 both have a Kozak sequence [20], the reason for the shift of the translation start may not necessarily lie in the primary sequence. Further, in the full-length AGO2 mRNA also both AUG are present within the same surrounding sequence, but the first one is more strongly used, suggesting that more global changes must contribute to the alternative translational start induced by the exclusion of exon2. Next to the primary sequence, the secondary structure around the start codon can significantly influence translation initiation [27]. Thereby, stable hairpin structures directly at the AUG or upstream can shield translation initiation whereas downstream secondary structures can support translational start when they force ribosome stalling near the start codon [27, 28]. It is conceivable that the AGO2-ex1/3 mRNA structure may be altered by the absence of exon 2 in such a way that the first AUG is obscured by a secondary structure, thereby preventing translation initiation.

Further, downstream translation initiation can be initiated 5´cap-independent by internal ribosome entry sites (IRES) [29]. Research in the recent years could show that this phenomenon, known from viral RNAs, can also occur in human mRNAs. The AGO2-ex1/3 sequence shares 85% identity to an IRES sequence also found in the gene MYCN (http://iresite.org/IRESite_web.php). In MYCN, this IRES enhances translation of N-myc especially in neuronal cell lines [30]. In AGO2-ex1/3, the sequence is located right after the first AUG in exon1. By skipping exon2, the IRES gets spatially close to the second AUG, which would be localized in the normal AGO2 mRNA 193 bp further downstream. This close spatial proximity might facilitate a translational start through this possible IRES sequence from this second AUG in AGO2-ex1/3.

Translation of AGO2-ex1/3 leads to a truncated AGO2 protein version missing the first 77 amino acids at the N-terminus, which are structurally located mainly in the N-domain of the AGO2 protein [22]. The N-domain is known to be important for the ability of AGO2 to unwind a bound miRNA or siRNA duplex, preparing the RISC for efficient target mRNA binding [31]. Kwak et al. could show that a deletion of the whole N-domain (amino acids 53–135) leads to an AGO2 version which can still load, but is unable to unwind small RNA duplexes [31]. They concluded that the N-domain plays a role as conformational wedge enforcing duplex unwinding after RISC loading [31]. Our data propose an increased flexibility especially of the N-domain in the AGO2-ex1/3 protein structure. It suggests that at least in part the unwinding function may be impaired, assuming that the limited flexibility of the N-domain in the full-length AGO2 structure is largely responsible for this wedging function during duplex unwinding.

Simulation of the AGO2-ex1/3 protein structure continues to show that the other AGO2 domains, PAZ, MID, and PIWI, are largely structurally unaffected by truncation of the first 77 amino acids. These data suggest that, besides duplex unwinding, other functions of AGO2, such as miRNA duplex loading or target mRNA binding and cleavage, could be retained in AGO2-ex1/3.

Further, the molecular dynamics simulation revealed an increased interdomain flexibility especially for the cleft between the N + PAZ- and the MID + PIWI-lobe of the AGO2-ex1/3 structure in which for AGO2 the guide miRNA strand is nested and target mRNA binding takes place [22]. This may open new functional possibilities for guide RNA to target RNA binding. It is known from the AGO2 structure, that the N-domain forms together with the PAZ domain a narrow channel in which the 3'-half of the guide miRNA is embedded [32]. The crystal structure of AGO2 bound to a guide miRNA suggests that this conformation prevents base pairing of the 3'-half of the miRNA with a complementary target [32]. In AGO2-ex1/3, a helix in the N-domain formed by amino acid P67-F82 is missing, which by its position must be crucially involved in the formation of the narrow N + PAZ-cleft. This suggests that the absence of amino acid 1–77 in AGO2-ex1/3 could result in a conformational change of the N-domain in a way that may be more conducive to complementary base pairing to the 3´ region of the guide miRNA.

For classical miRNA-mediated gene regulation, which means complementary base pairing to the 3'-UTR of target mRNAs, a binding of the so called “seed” region, nucleotide 2–8 of the guide miRNA, plays a particularly important role [4]. Recent data in the literature show that non-canonical 3'-miRNA-base pairing can also be important for the regulation of target mRNAs, especially for regulation in the coding sequence [33].

In our study, we observed a strong reduction of melanoma cell growth after knockdown of AGO2-ex1/3. Our data indicate that the reason for this reduced growth may be related in large proportion, but probably not exclusively, to apoptosis. RNA-Seq after AGO2-ex1/3 knockdown revealed no significant changes in specific molecular signaling pathways, but a strong upregulation of a large variety of miRNA target gene sets. Some of the identified, upregulated genes are described in the literature as associated to apoptotic mechanisms. E.g., ACVR1C (also activin receptor-like kinase, ALK7), a receptor for transforming growth factor (TGF) β signaling molecules, can be overexpressed in a constitutively active version, induce apoptosis in hepatocellular carcinoma via activation of caspase3 in a Smad3-dependent manner [34]. Or the desumoylating isopeptidase 2 (DESI2), which induces apoptosis in lung adenocarcinoma cells when overexpressed [35]. In pancreatic cancer cells, DESI2 overexpression leads to upregulation of Ras homolog gene family member A (RhoA) and Rho‑associated protein kinase, proteins which are known to be involved in stress fiber formation [36].

However, as our study revealed the upregulation of a large variety of target genes of many different miRNAs, we conclude that the observed reduced cell growth after AGO2-ex1/3 knockdown may not be due to mis-regulation of only one single signaling pathway. The upregulation of multiple different miRNA target genes indicates that the AGO2-ex1/3 knockdown must lead to a more global impact on general miRNA function. Thus, AGO2-ex1/3 seems to have a strong impact on miRNA abundance or on RISC activity. Due to a defect in miRNA regulation, numerous target genes involved in various cellular processes are aberrantly expressed, which ultimately leads to the observed apoptosis after AGO2-ex1/3 knockdown in melanoma cells. However, to examine which molecular role AGO2-ex1/3 plays exactly for miRNA processing and function and to obtain a more detailed picture of the functional differences between AGO2 and AGO2-ex1/3, further studies will be needed.

The data revealed in this study indicate a growth advantage of melanoma cells expressing a previously unknown AGO2 variant and a potential functional significance of this variant for the miRNA pathway. This study gives important insights into a yet unknown molecular mechanism regarding miRNA function in melanoma cells. It makes a substantial contribution to a better understanding of the complex molecular processes of melanoma development and malignancy on one hand as well as of miRNA-mediated gene regulation on the other hand. Both build an important basis for the development of potential future miRNA-based molecular therapy approaches for malignant melanoma.

## Supplementary Information

Below is the link to the electronic supplementary material.

Supplementary file1 (PDF 2398 KB)

Supplementary file2 (PDF 1759 KB)

Supplementary file3 (PDF 463 KB)

Supplementary file4 (XLSX 5296 KB)

## Data Availability

The RNA-sequencing data used in this study have been deposited in the NCBI BioProject database (https://www.ncbi.nlm.nih.gov/bioproject/) and can be accessed with the BioProject accession number PRJNA801437.
