# Supplementary material for: A previously unknown Argonaute 2 variant positively modulates the viability of melanoma cells
Source: Cell Mol Life Sci. 2022 Aug 9;79(9):475. doi: 10.1007/s00018-022-04496-8 (PMC9363364; doi:10.1007/s00018-022-04496-8)
Supplement: Supplementary file 1 — Supplementary file1 (PDF 2398 KB) [file 18_2022_4496_MOESM1_ESM.pdf]

Supplementary File 1

Supplementary figures S1-S9

A previously unknown Argonaute 2 variant positively modulates the viability of melanoma cells

Lisa Linck-Paulus, Tina Meißgeier, Katharina Pieger, Anselm H.C. Horn, Alexander Matthies, Stefan Fischer, Gunter Meister, Heinrich Sticht, Melanie Kappelmann-Fenzl and Anja Katrin Bosserhoff

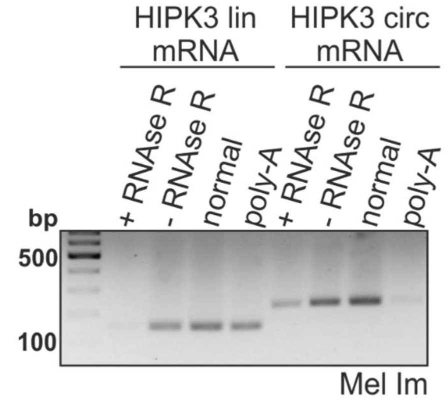

Supplementary Figure S1: PCR amplification of linear and circular HIPK3.
PCR products of linear (lin) and circular (circ) HIPK3 after amplification of RNase R digested RNA, control RNA without RNase R, normally transcribed RNA and RNA transcribed with a poly(A)-specific primer in the melanoma cell line Mel Im. The amplification with primers targeting HIPK3 lin showed no product for the sample treated with RNase R, but a band is visible for samples without RNAse R or for normally transcribed cDNA as well as for the poly(A)-specific cDNA (lane 1-4). For HIPK3 circ a product occurs after RNase R treatment, but no band can be observed in the poly(A)-sample (lane 5-8).

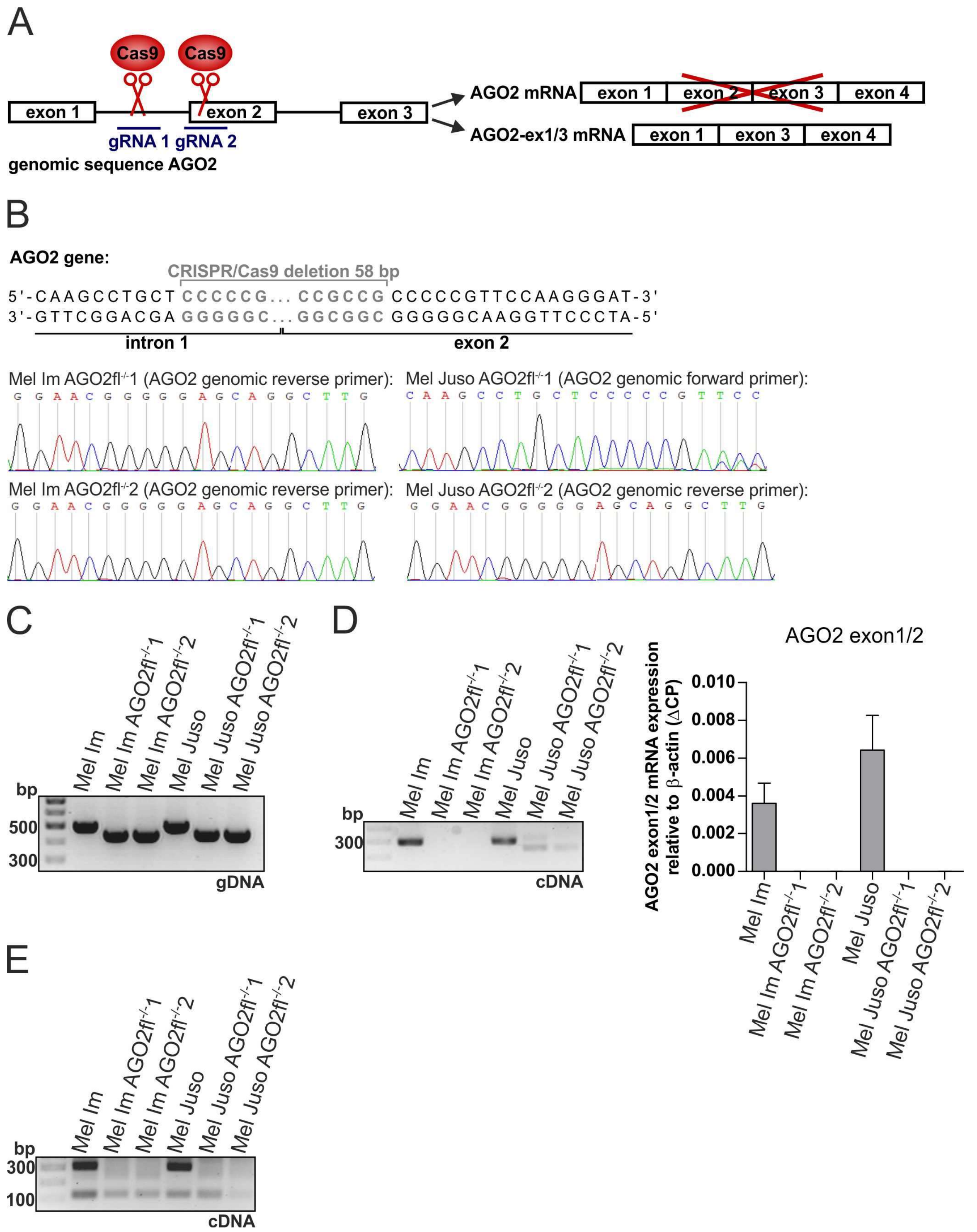

Supplementary Figure S2: CRISPR-Cas9-mediated knockout of AGO2 in melanoma cells.

(**A**) Scheme of the CRISPR/Cas9 mediated knockout of AGO2 using two guide RNA sequences (gRNA1+2) leading to a genomic deletion which prevents insertion of exon2 into the AGO2 mRNA. The knockout strategy was derived from van Eijl et al. (19). (**B**) DNA sequencing of the AGO2 knockout melanoma cell lines. The scheme represents the CRISPR/Cas9-mediated deletion in the AGO2 gene. (**C**) PCR products after amplification of genomic DNA (gDNA) of the AGO2 full length knockout cell lines Mel Im AGO2fl$^{-/-}$ 1+2 and Mel Juso AGO2fl$^{-/-}$ 1+2 and the respective wild type cell lines using AGO2 genomic primers spanning the CRISPR/Cas9 targeted 58 bp deletion site. (**D**) QRT-PCR-amplification of cDNA from the CRISPR/Cas9-mediated AGO2 knockout clones Mel Im AGO2fl$^{-/-}$ 1+2 and Mel Juso

AGO2fl$^{-/-}$ 1+2 and the respective wild type cell lines using primers specific for the transition between AGO2 exon1 and exon2 (mean±SD from n=3-6). (**E**) PCR products after amplification of AGO2 exon 1-3 with cDNA of the AGO2 full length knockout cell lines Mel Im AGO2fl$^{-/-}$ 1+2 and Mel Juso AGO2fl$^{-/-}$ 1+2 and the respective wild type cell lines.

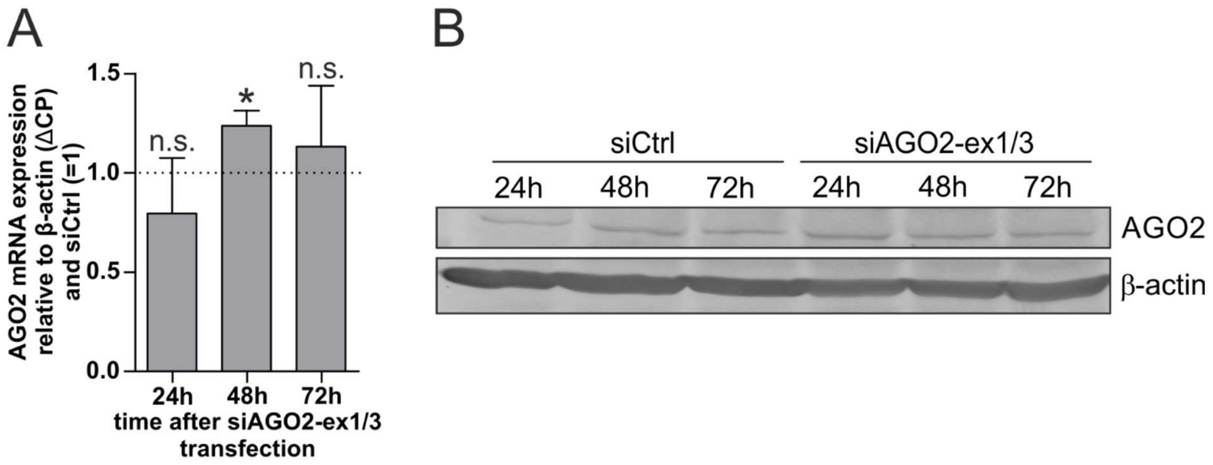

Supplementary Figure S3: SiAGO2-ex1/3 does not reduce full length AGO2 expression. (**A**) Relative AGO2 mRNA expression to β-actin (ΔCP) and siCtrl (=1), analyzed via qRT-PCR in the melanoma cell line Mel Im after 24, 48 or 72 h transfection with siAGO2-ex1/3 or a control siRNA (mean±SD from n=4 (24h, 48h) or n=11 (72h), *=p<0.05, n.s.=not significant, one sample t-test compared to 1). (**B**) Western blot stained with antibodies against AGO2 (N-terminal epitope) and β-actin (loading control) of Mel Im transfected as described in A.

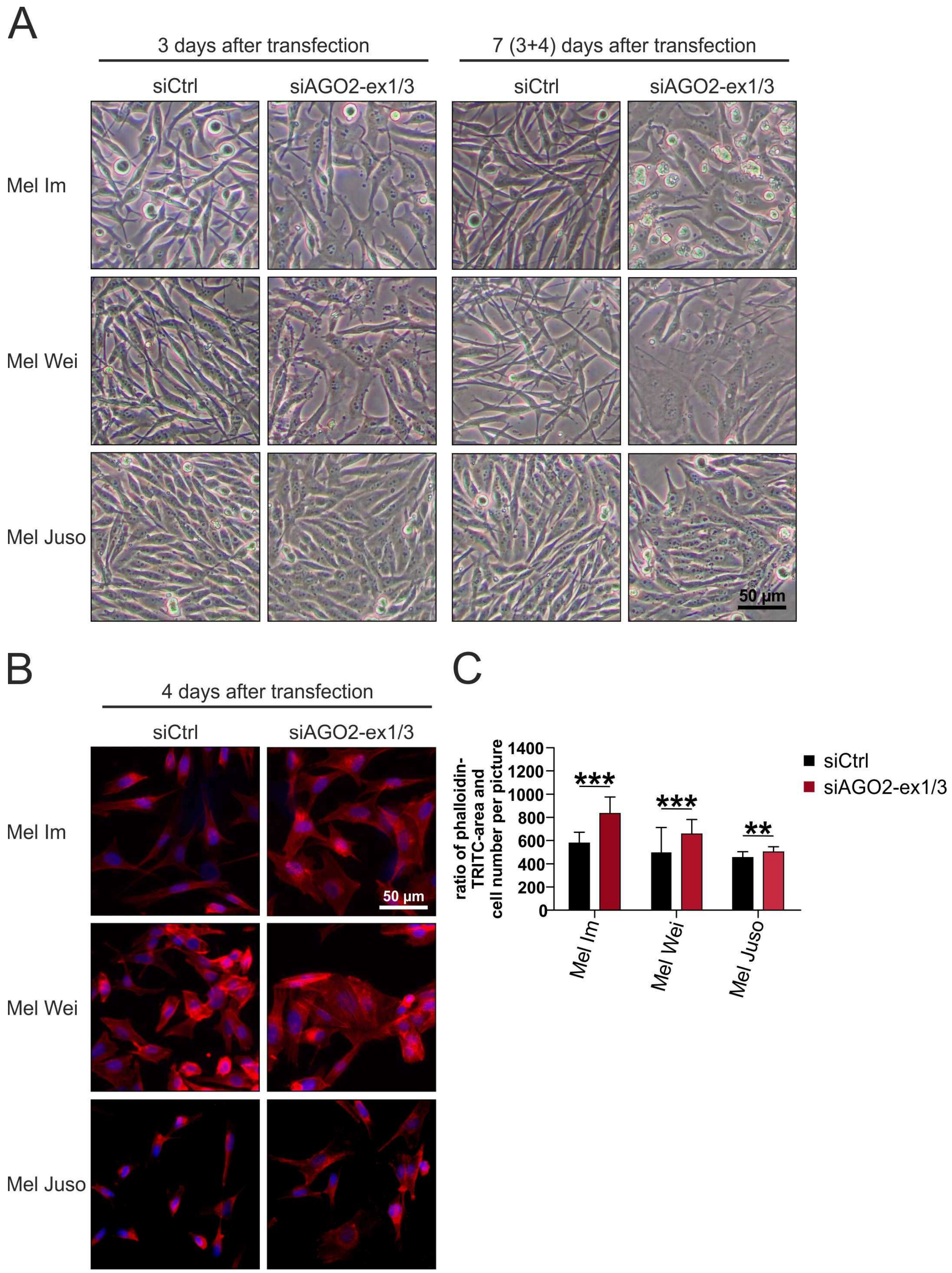

Supplementary Figure S4: Changes in cell morphology after AGO2-ex1/3 knockdown. (**A**) Light microscopy images of Mel Im, Mel Wei and Mel Juso melanoma cell lines after 3 days or 7 days (with re-transfection at day 3) transfection with siAGO2-ex1/3 or siCtrl (**B**) SiRNA transfected melanoma cells were stained for filamentous actin using phalloidin-TRITC (red) and cell nuclei with DAPI (blue). (**C**) Quantification of phalloidin-TRITC area relative to the number of nuclei per image with at least 10 images each from 3 independent experiments. Graphs show mean ±SD (**=p<0.01 and ***=p<0.001, unpaired t-test).

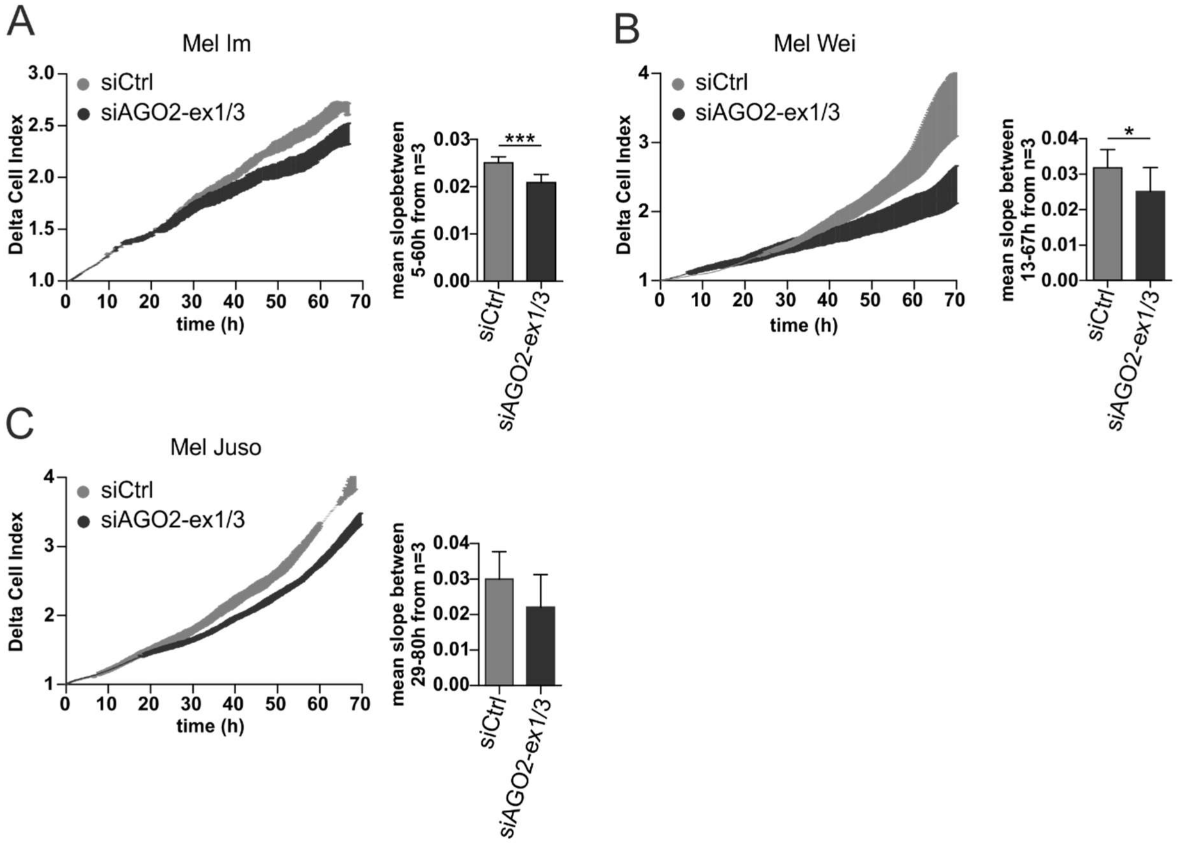

Supplementary Figure S5: Knockdown of AGO2-ex1/3 reduces cell growth in real time cell analysis.

Real time cell analysis (RTCA) showing the Delta Cell Index of the melanoma cell lines (**A**) Mel Im, (**B**) Mel Wei and (**C**) Mel Juso after transfection with siAGO2-ex1/3 or a control siRNA. The assay starts 4 days after transfection (with re-transfection at day 3). Graphs show one exemplarily experiment with the SD-range of 2-3 technical replicates. Bars show mean slope of the delta cell index ±SD in a cell line specific time range from n=3 (*=p<0.05 and ***=p<0.001, unpaired t-test).

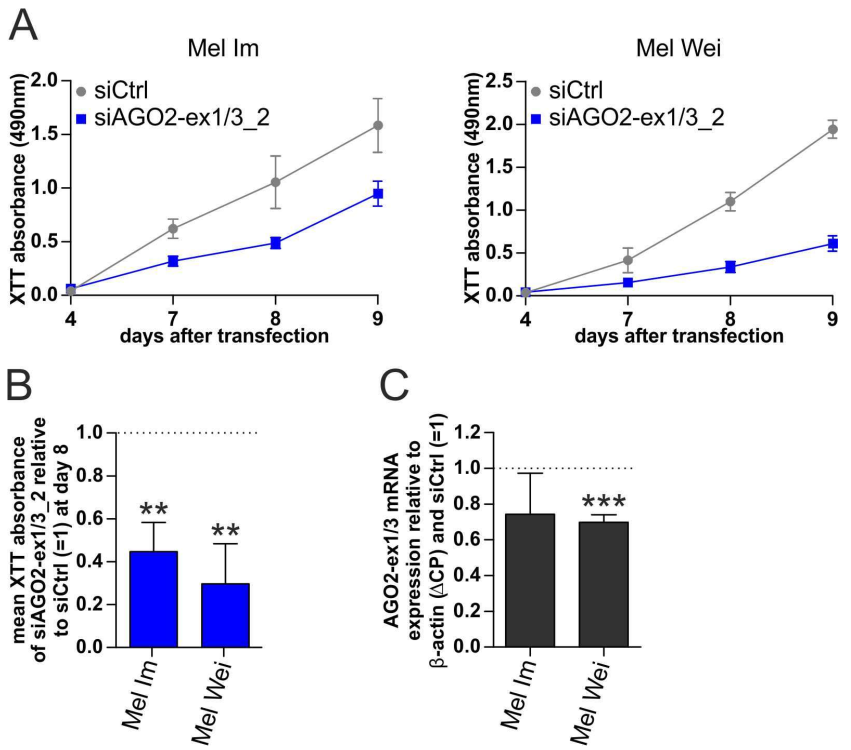

Supplementary Figure S6: Knockdown of AGO2-ex1/3 using a second, specific siRNA (siAGO2-ex1/3_2), reduces cell proliferation.
(**A**) XTT viability assay of the melanoma cell lines Mel Im and Mel Wei showing XTT absorbance at displayed time points after transfection with siAGO2-ex1/3_2 or a control siRNA (with re-transfection at day 3). Graphs show one exemplarily assay for each cell line with three technical replicates. (**B**) Mean absorbance values of siAGO2-ex1/3_2-treated relative to siCtrl-treated cells (=1) at day 8 (mean±SD from n=4, *=p<0.05, **=p<0.01, one sample t-test compared to 1). (**C**) Relative AGO2-ex1/3 mRNA expression to β-actin (ΔCP) and siCtrl (=1), analyzed via qRT-PCR in the indicated melanoma cell lines after 3+4 days transfection with siAGO2-ex1/3_2 (mean±SD, n=4, ***=p<0.001, one sample t-test compared to 1).

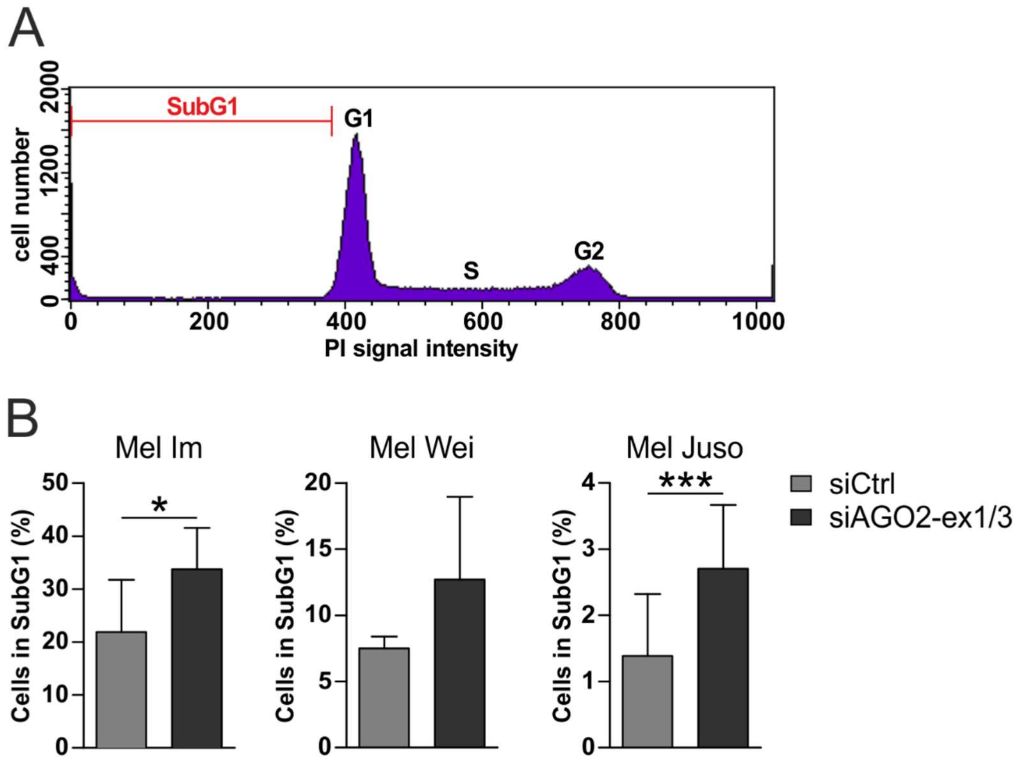

Supplementary Figure S7: Increase in SubG1 cells after siAGO2-ex1/3 transfection.
(**A**) Exemplary gating of potential apoptotic SubG1 cells in Mel Im using propidium iodide (PI) signal intensity and flow cytometry. (**B**) Average cell number assigned to SubG1 (%) from PI staining of the indicated melanoma cell lines transfected for 7 days (with re-transfection at day 3) with si-AGO2-ex1/3 or a control siRNA (mean±SD, n=5 (Mel Im), n=3 (Mel Wei, Mel Juso), *=p<0.05,***=p<0.001, paired t-test).

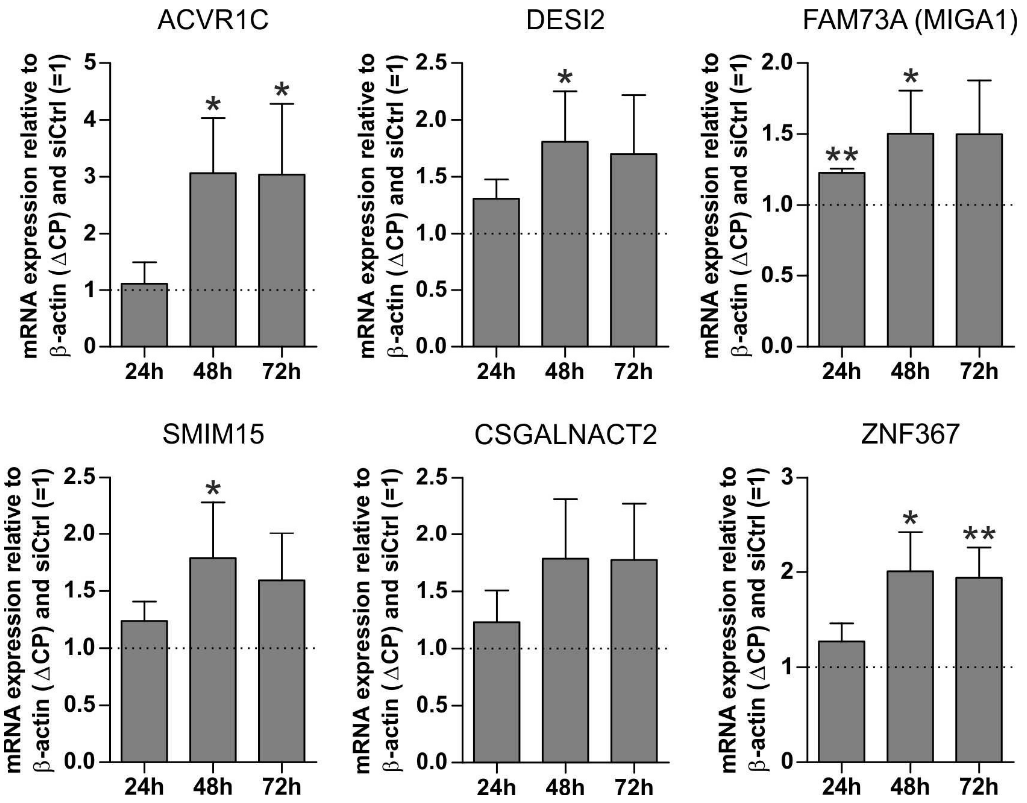

Supplementary Figure S8: Validation of increased expression of several miRNA target genes after knockdown of AGO2-ex1/3.

Relative mRNA expression of indicated genes to β-actin (ΔCP) and siCtrl (=1), analyzed via qRT-PCR in the melanoma cell line Mel Juso after 24, 48 or 72 h transfection with siAGO2-ex1/3 or a control siRNA (mean±SD from n=3 (24h) or n=4 (48, 72h), *=p<0.05, **=p<0.01, one sample t-test compared to 1).

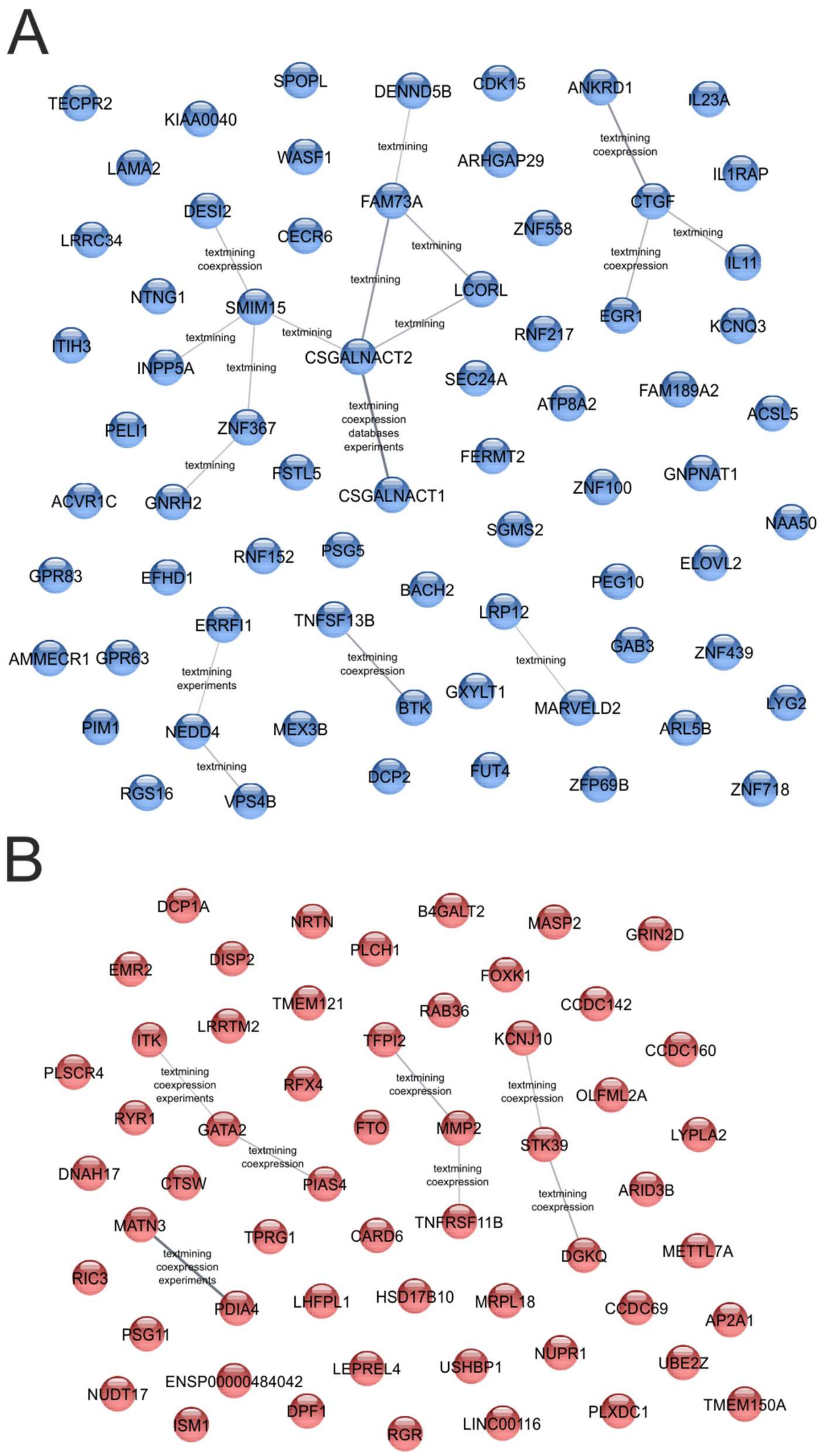

Supplementary Figure S9: Genes affected by AGO2-ex1/3 knockdown do not belong to a certain molecular network.

STRING protein network analysis of strongly (log2 FoldChange >0.585 or <-0.585 corresponds to more than 1.5 fold) and significantly ($p<0.05$) upregulated (**A**) or downregulated (**B**) genes in siAGO2-ex1/3- compared to siCtrl-treated cells in the cell line Mel Juso. Interactions based on textmining, coexpression, databases or experimental evidence are indicated. The proteins show no significant pathway enrichments in Gene Ontology (Biological Process, Molecular

Function or Cellular Component), KEGG Pathways, Reactome Pathways, WikiPathways, Disease-gene associations, Tissue expression, Subcellular localization, UniProt Annotated Keywords, Pfam Protein Domains, InterPro Protein Domains and Features or SMART Protein Domains according to the STRING analysis.
