# Supplementary material for: A previously unknown Argonaute 2 variant positively modulates the viability of melanoma cells
Source: Cell Mol Life Sci. 2022 Aug 9;79(9):475. doi: 10.1007/s00018-022-04496-8 (PMC9363364; doi:10.1007/s00018-022-04496-8)
Supplement: Supplementary file 2 — Supplementary file2 (PDF 1759 KB) [file 18_2022_4496_MOESM2_ESM.pdf]

Supplementary File 2

Detailed description of the molecular dynamics simulations of the AGO2-ex1/3 protein structure

A previously unknown Argonaute 2 variant positively modulates the viability of melanoma cells

Lisa Linck-Paulus, Tina Meißgeier, Katharina Pieger, Anselm H.C. Horn, Alexander Matthies, Stefan Fischer, Gunter Meister, Heinrich Sticht, Melanie Kappelmann-Fenzl and Anja Katrin Bosserhoff

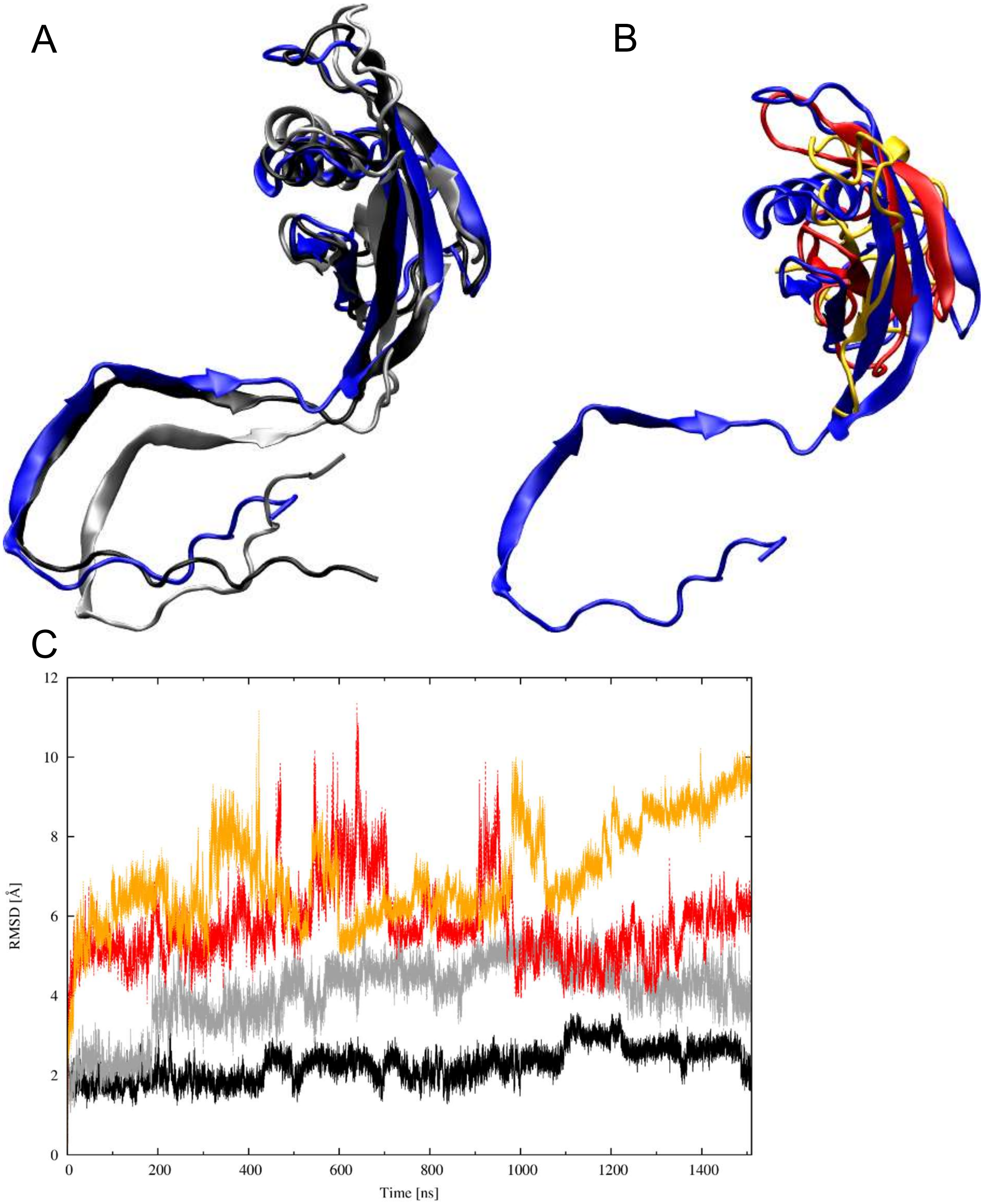

Figure I: **N-domain (residue 53-139) flexibility in molecular dynamics simulation.**
(**A**) Superimposition of the crystal structure (**blue**) with the final structures of the two simulations with full-length AGO2 (**black**, **grey**). (**B**) Superimposition of the crystal structure (**blue**) with the final structures of the two simulations with truncated AGO2-ex1/3 (**red**, **orange**). (**C**) RMSD evolution during the simulations (color coding is according to **A**, **B**).
While in full-length AGO2 the N-domain kept its overall structure, in the truncated species AGO2-ex1/3 a partial unfolding was observed. The stronger increase in RMSD for AGO2-ex1/3 indicates that truncation leads to a larger structural disturbance.

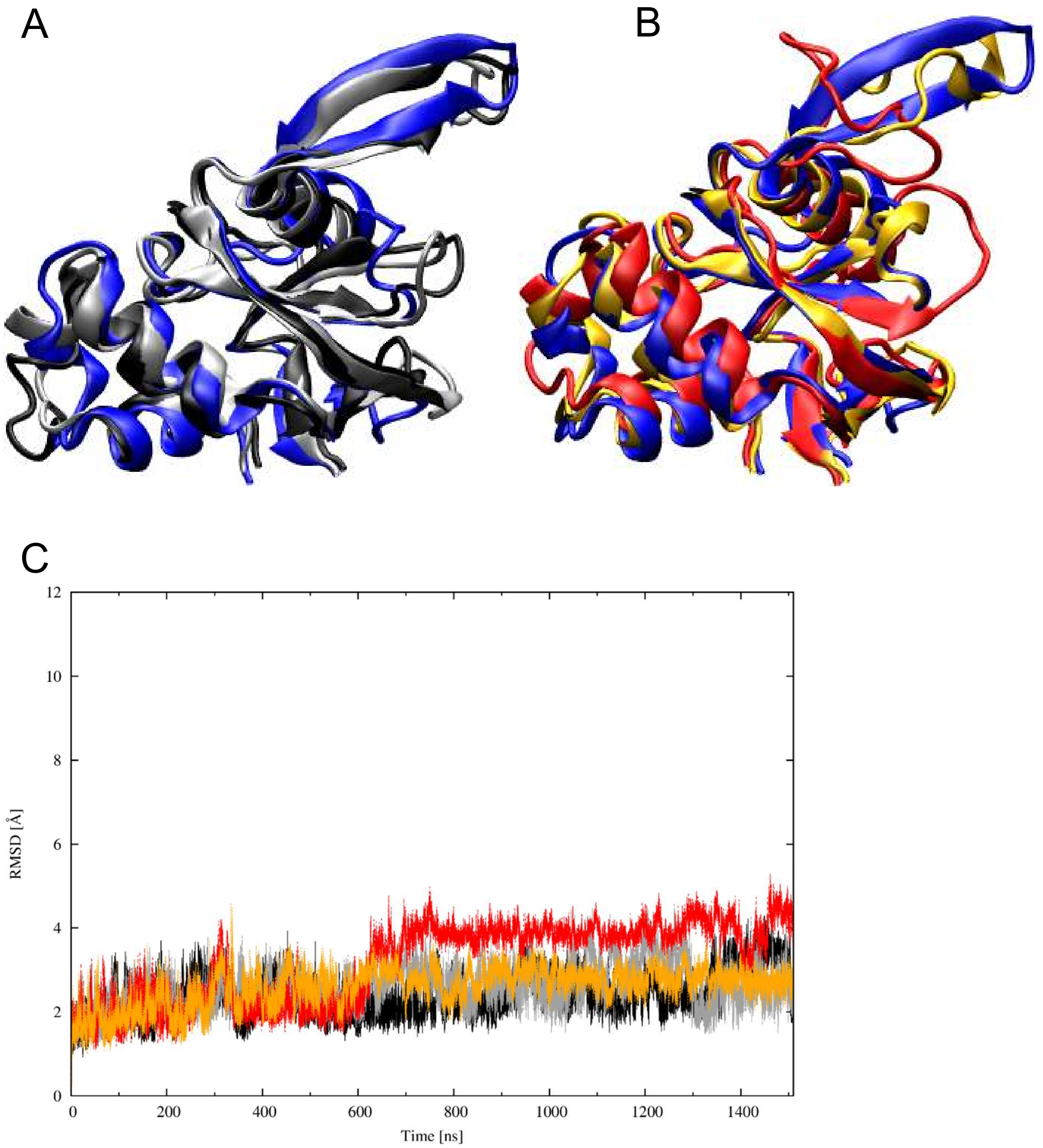

Figure II: **PAZ domain (residue 229-347) flexibility in molecular dynamics simulation.** (**A**) Superimposition of the crystal structure (**blue**) with the final structures of the two simulations with full-length AGO2 (**black**, **grey**). (**B**) Superimposition of the crystal structure (**blue**) with the final structures of the two simulations with truncated AGO2-ex1/3 (**red**, **orange**). (**C**) RMSD evolution during the simulations (color coding is according to **A**, **B**).

The overlay and similar RMSD values show that the structure of the PAZ domain did not change significantly.

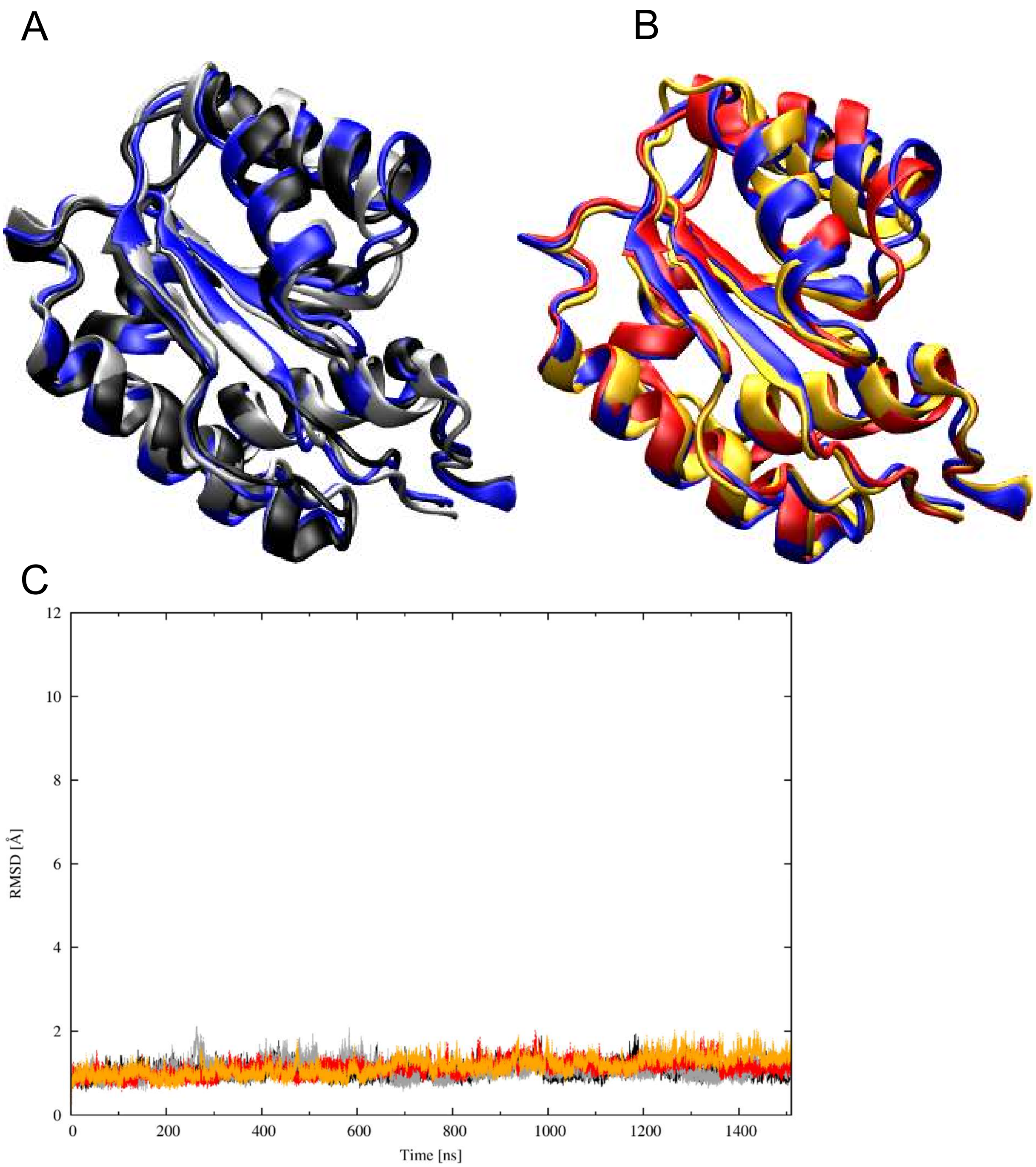

Figure III: **MID domain (445-577) flexibility in molecular dynamics simulation.**
(**A**) Superimposition of the crystal structure (**blue**) with the final structures of the two simulations with full-length AGO2 (**black**, **grey**). (**B**) Superimposition of the crystal structure (**blue**) with the final structures of the two simulations with truncated AGO2-ex1/3 (**red**, **orange**). (**C**) RMSD evolution during the simulations (color coding is according to **A**, **B**). Clearly, the final structures of all four simulations were nearly identical to the initial structure.
The RMSD graph shows almost a constant value over the simulation course indicating that was retained during the simulation in the full-length and truncated protein.

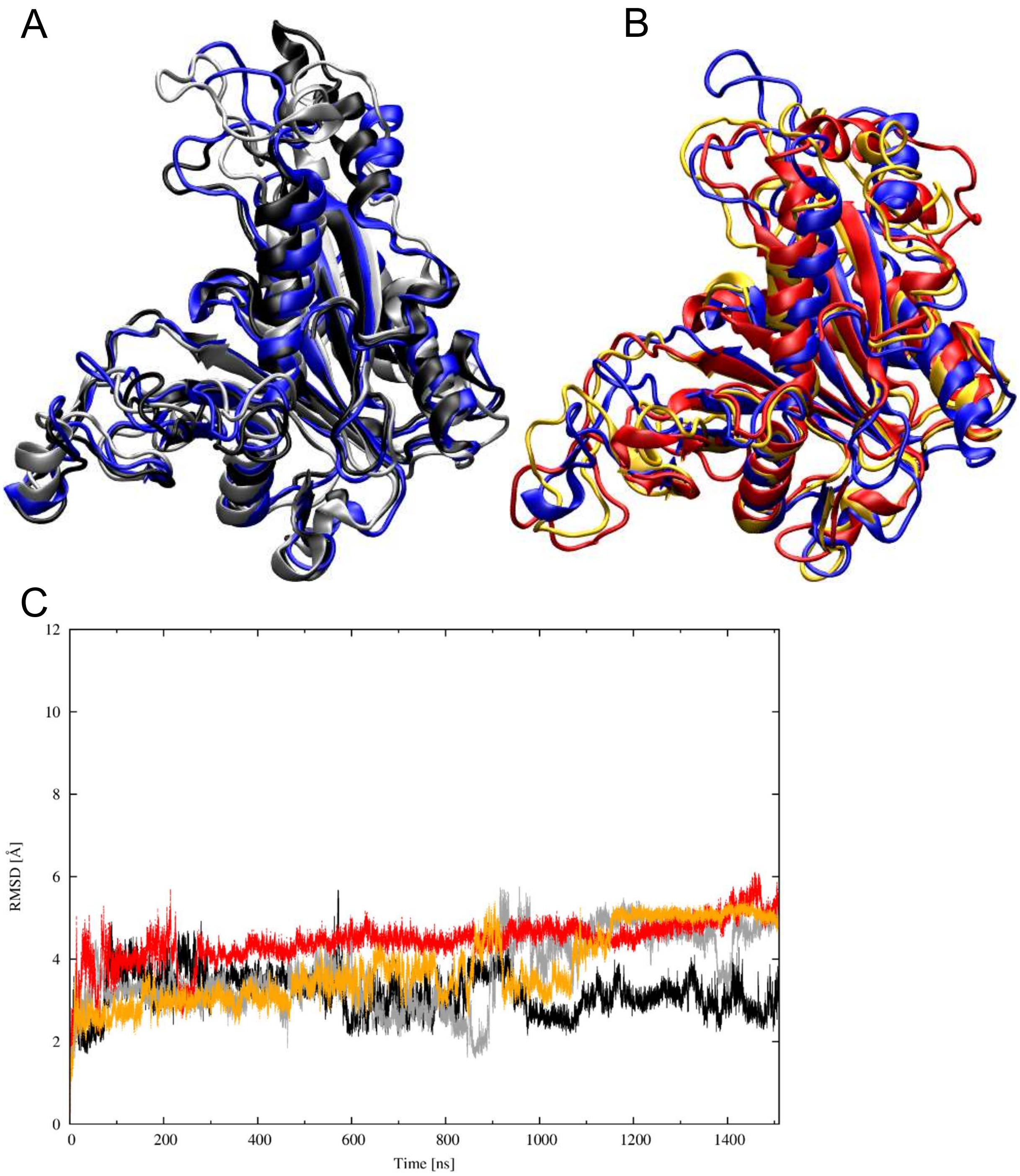

Figure IV: **Piwi domain (578-859) flexibility in molecular dynamics simulation.**
(**A**) Superimposition of the crystal structure (**blue**) with the final structures of the two simulations with full-length AGO2 (**black**, **grey**). (**B**) Superimposition of the crystal structure (**blue**) with the final structures of the two simulations with truncated AGO2-ex1/3 (**red**, **orange**). (**C**) RMSD evolution during the simulations (color coding is according to **A**, **B**).
The final structures of all four simulations remain similar to the initial structure. As a general feature of the intact and truncated AGO2, a rather high flexibility of the loops in the Piwi domain is detected. The RMSD graph shows similar low values for all four simulations over the simulation time indicating an intact domain architecture. The larger absolute values compared to the MID domain (**Figure III**) can be attributed to the more flexible loop regions.

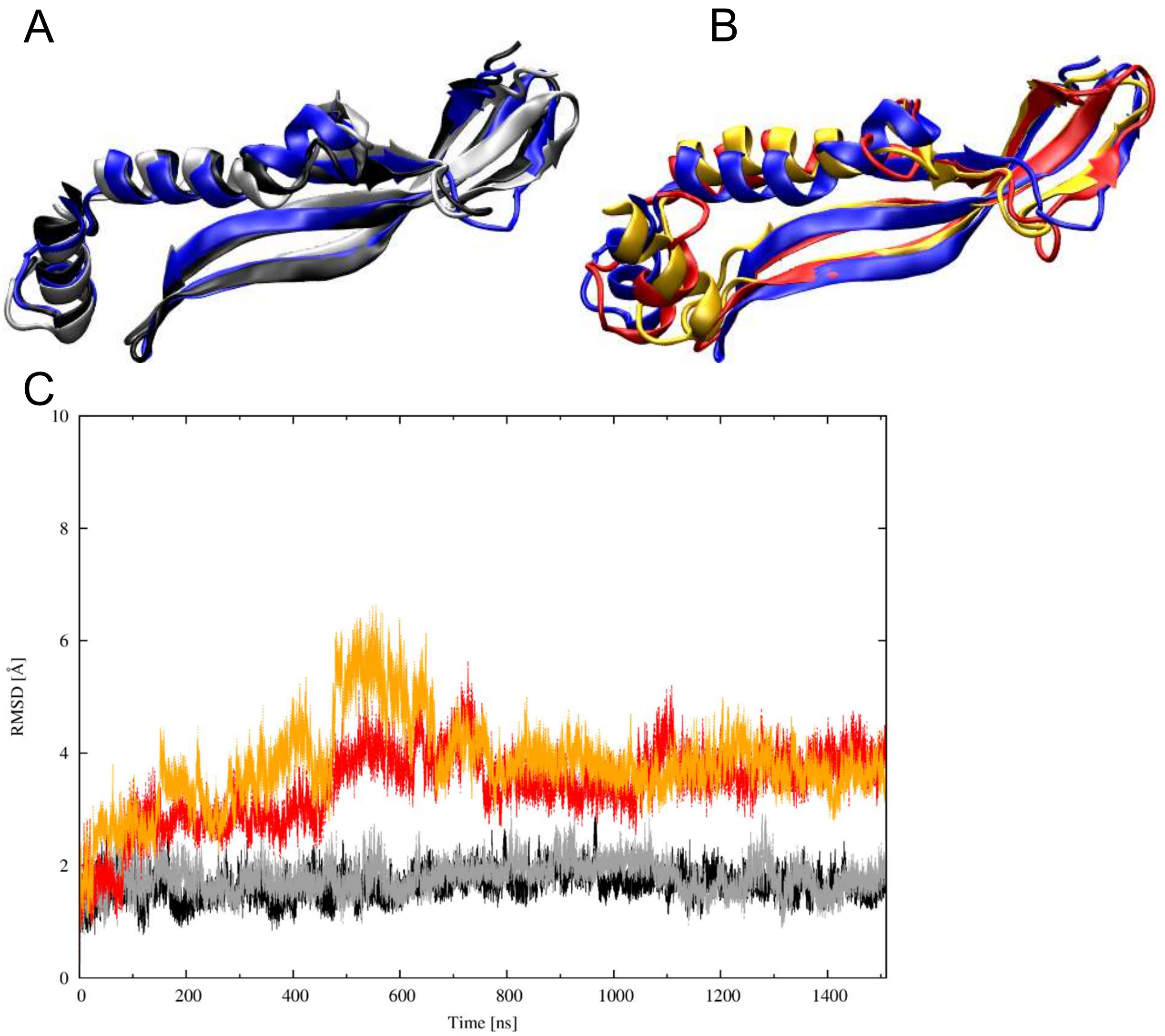

Figure V: **L1 linker domain (residue 140-228) flexibility in molecular dynamics simulation.**
(**A**) Superimposition of the crystal structure (**blue**) with the final structures of the two simulations with full-length AGO2 (**black**, **grey**). (**B**) Superimposition of the crystal structure (**blue**) with the final structures of the two simulations with truncated AGO2-ex1/3 (**red**, **orange**). (**C**) RMSD evolution during the simulations (color coding is according to **A**, **B**). Obviously, the depicted structures in the full-length AGO2 species were nearly identical to the initial structure, while the truncated AGO2 species displayed a higher flexibility (e.g. in the short helix motif). The RMSD graph for full-length AGO2 shows almost a constant value for the L1 linker domain over the simulation time indicating a stable domain structure. In the truncated AGO2-ex1/3 species, this linker domain constantly displays a slightly higher flexibility.

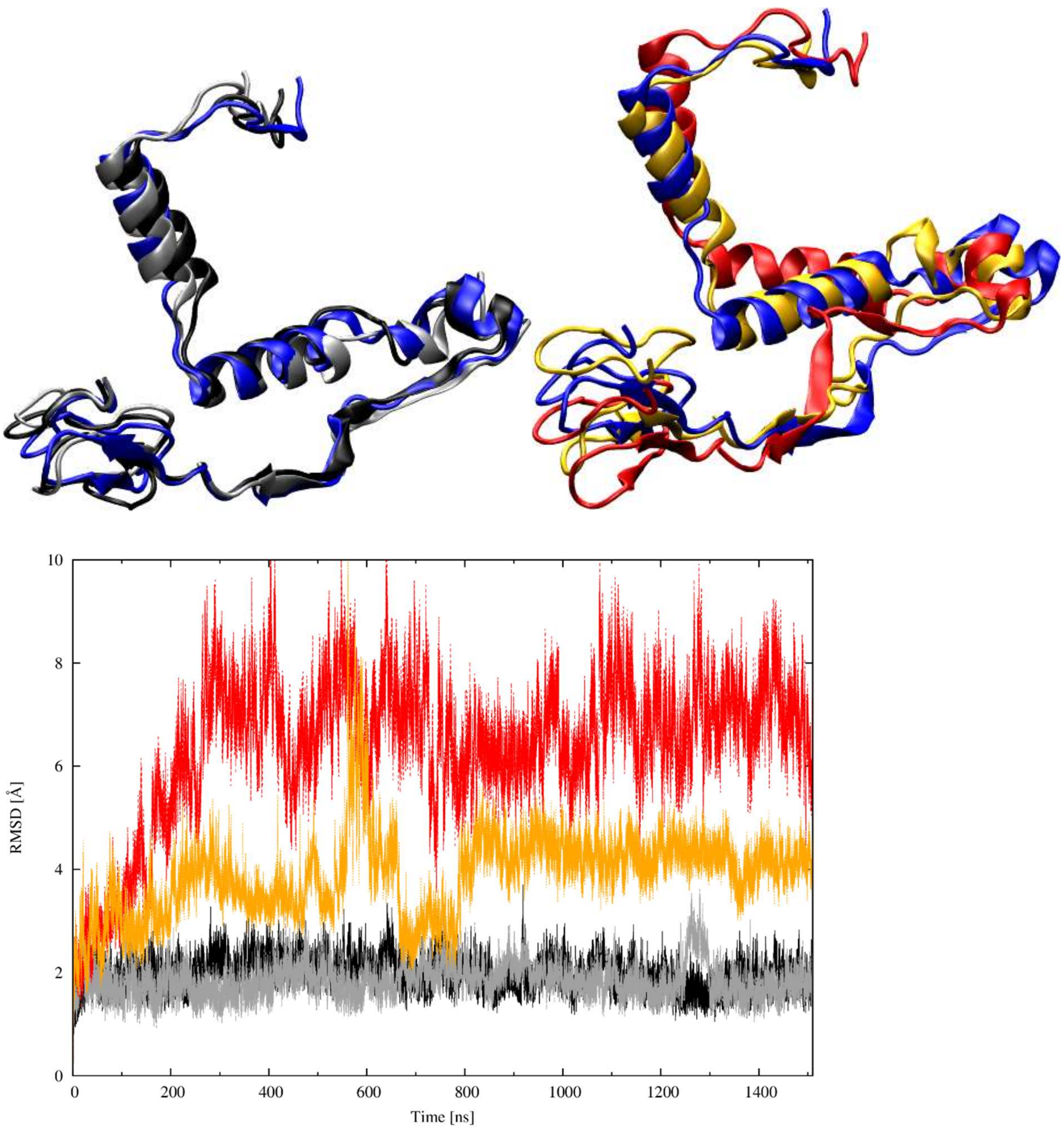

Figure VI: **L2 linker domain (residue 348-444) flexibility in molecular dynamics simulation.**
(**A**) Superimposition of the crystal structure (**blue**) with the final structures of the two simulations with full-length AGO2 (**black**, **grey**). (**B**) Superimposition of the crystal structure (**blue**) with the final structures of the two simulations with truncated AGO2-ex1/3 (**red**, **orange**). (**C**) RMSD evolution during the simulations (color coding is according to **A**, **B**).
The depicted structures show a similar yet more pronounced trend as for the L1 linker region: while the L2 domain in the full-length AGO2 species were nearly identical to their initial structure, this linker domain displayed a significantly enhanced flexibility in the truncated AGO2 species, especially for the C-terminal residues.

A

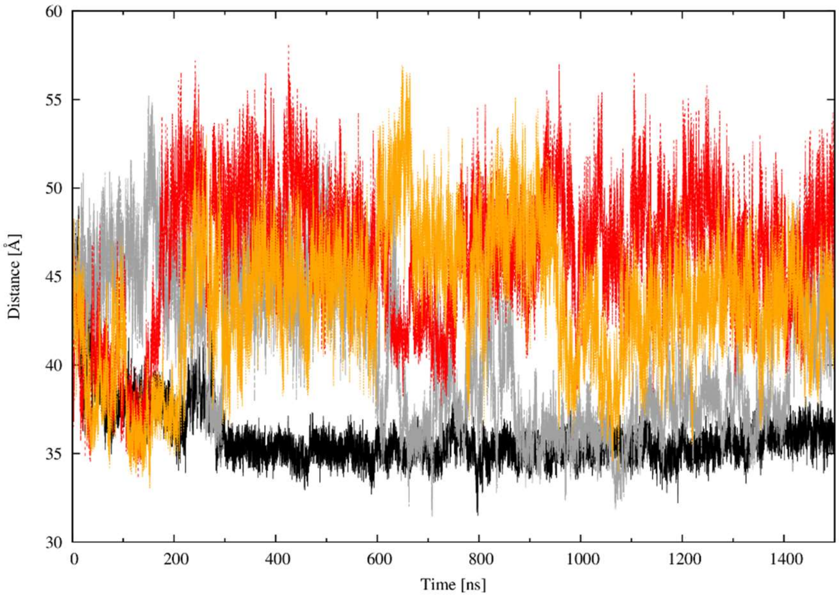
Distance [Å]
Time [ns]
60
55
50
45
40
35
30
0
200
400
600
800
1000
1200
1400

B

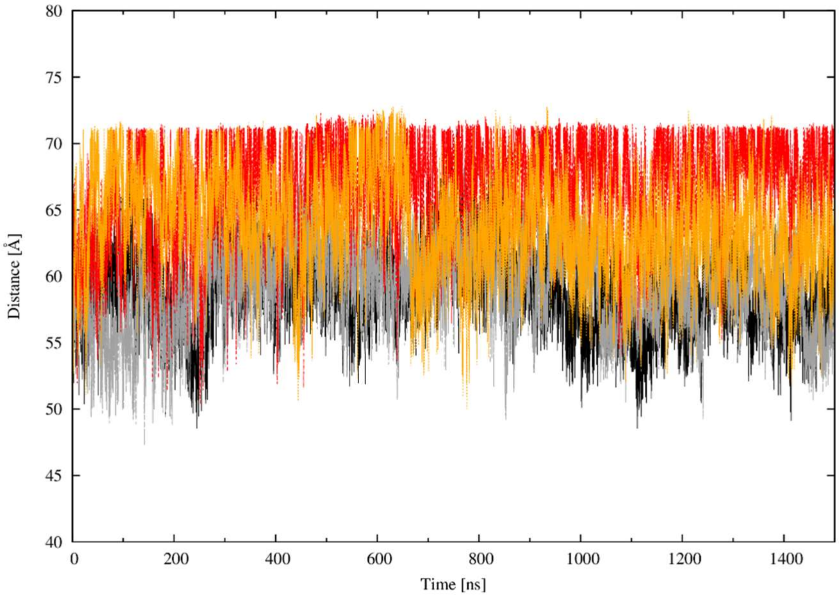
Distance [Å]
Time [ns]
80
75
70
65
60
55
50
45
40
0
200
400
600
800
1000
1200
1400

C

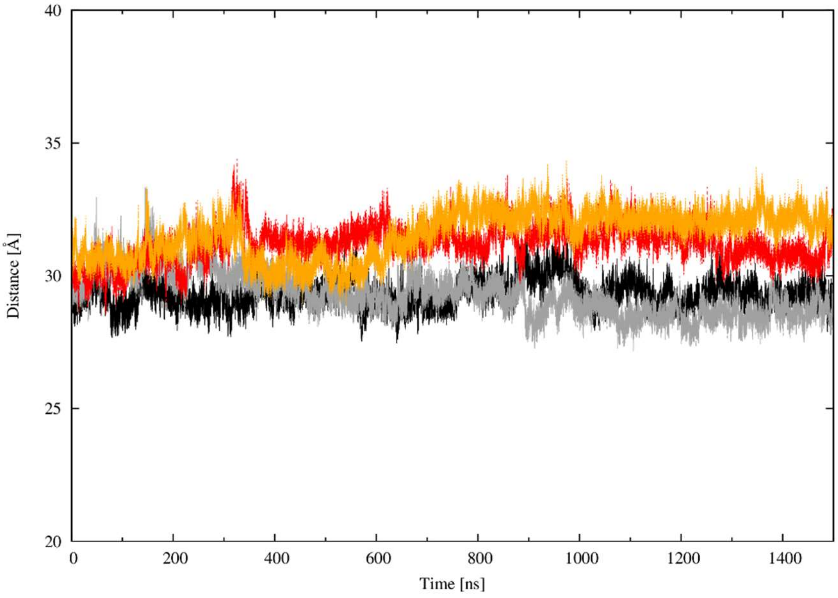
Distance [Å]
Time [ns]
40
35
30
25
20
0
200
400
600
800
1000
1200
1400

Figure VII: **Inter-domain distances measured between the center of mass of two different domains.**
Values for full-length AGO2 are in **black** and **grey**, values for truncated AGO2-ex1/3 in **red** and **orange**.
(**A**) Distance between N-domain and PAZ domain.
(**B**) Distance between PAZ and MID domain.
(**C**) Distance between MID and Piwi domain.
The influence of the truncation on the inter-domain flexibility is most pronounced for the orientation of the N-domain and PIWI domain (**A**), where the distance shows variations of ca. 15 Å. The truncation has a remote effect on the distance between the PAZ and MID domain (**B**), although to a lesser extent. The domain pair MID-Piwi is not significantly influenced by the truncation, and the domain-domain distance is only slightly increased (**C**).
