# Supplementary material for: A previously unknown Argonaute 2 variant positively modulates the viability of melanoma cells
Source: Cell Mol Life Sci. 2022 Aug 9;79(9):475. doi: 10.1007/s00018-022-04496-8 (PMC9363364; doi:10.1007/s00018-022-04496-8)
Supplement: Supplementary file 3 — Supplementary file3 (PDF 463 KB) [file 18_2022_4496_MOESM3_ESM.pdf]

Supplementary File 3

Additional descriptions of materials and methods

A previously unknown Argonaute 2 variant positively modulates the viability of melanoma cells

Lisa Linck-Paulus, Tina Meißgeier, Katharina Pieger, Anselm H.C. Horn, Alexander Matthies, Stefan Fischer, Gunter Meister, Heinrich Sticht, Melanie Kappelmann-Fenzl and Anja Katrin Bosserhoff

Immunofluorescence

Melanoma cells were seeded into 12-wells supplemented with Ø 18 mm glass cover slips, grown over night and transfected with plasmid for 24 h; 72 h-siRNA transfected cells were seeded in 8 chamber polystryrene culture slides (Corning Inc., Corning, NY, USA) and grown for additional 24 h. Afterwards, cells were fixed with 4% paraformaldehyde in PBS for 15 min. All steps were performed at room temperature. Permeabilization was done with 0.1% Triton-X100 in PBS for 3 min and blocking with 10% bovine serum albumin (BSA, „heat shock fraction", pH 7, Sigma-Aldrich Chemie GmbH, Steinheim, Germany) in PBS for 30 min. Staining with phalloidin-TRITC (027PHDR1, 1:143 solution in PBS, Tebu-bio GmbH, Offenbach, Germany) was performed for 45 min. Afterwards, the cells were stained with 4′,6-Diamidin-2-phenylindol (DAPI, dilactate, 1:10,000 solution in PBS, Sigma-Aldrich Chemie GmbH, Steinheim, Germany) for 30 min. Subsequently, the slides were mounted using "Aqua-Poly/Mount" (Polysciences, Inc., Warrington, PA, USA), images were taken with the fluorescence microscope "IX83" (Olympus K.K., Shinjuku, Tokyo, Japan) with 20x magnification.

XTT viability assay

Cell proliferation and viability was determined using the "Cell Proliferation Kit II (XTT)" (Roche Diagnostics GmbH, Mannheim, Germany). The assay was started one day after re-transfection with siRNA. 1000 (Mel Im, Mel Wei) or 1600 (Mel Juso) transfected cells were seeded in triplicates into a 96-well plate in 100 µl medium without phenol red (PAN-Biotech GmbH, Aidenbach, Germany). The "XTT labeling mixture" was added at day 1, 4, 5, 6 and 7 to adherent cells according to the manufacturer's instructions and measured 4 h after adding the reagent at a "CLARIOStar" plate reader (BMG Labtech GmbH, Ortenberg, Germany) at 490 nm.

Clonogenic assay

For the clonogenic assay, 200 (Mel Wei) or 500 (Mel Im, Mel Juso) cells were seeded in a 6-well plate. After seven days, the cells were fixed and stained with 300 µl of a crystal violet solution (23,8 % glutaraldehyde, 36,2 % crystal violet, 10 % PBS). Each condition in each experiment was performed in two replicate wells. The number of colonies relative to the number of seeded cells was determined for each well with the software "CellSens Dimension" (Olympus K.K., Shinjuku, Tokyo, Japan) using color intensity, hue, and saturation. Colony size was defined as cell clusters of at least 40 pixel² (corresponds to approx. 50 cells).

Real time cell analysis (RTCA)

For RTCA, the "xCELLigence RTCA SP" real time cell analyzer (ACEA Biosciences Inc., San Diego, CA, USA) was used. For the analysis, 3000 (Mel Im, Mel Wei) or 4800 (Mel Juso) transfected cells in a volume of 100 µl medium were seeded in triplicates into an "E-Plate 96"

(ACEA Biosciences Inc., San Diego, CA, USA). The measurement of the "Cell Index" (CI) was performed every 5 min over a period of 8 h and for further 70 h with measurements every 15 min. The CI at a measurement time point x is calculated from the difference of the electrical alternating current resistance (impedance) on the sensor surface at this time and the reference resistance (preincubated culture medium without cells), divided by 15 Ω. For determination of the “Delta Cell Index”, the CI at a reference time is subtracted from the reference value 1 and this value is added to the respective CI for each measurement time point. The reference time point was determined individually for each cell line as time point where cell proliferation starts and is set to 0 h and “Delta Cell Index”=1 in the graphs.

Determination of senescence-associated β-galactosidase (SA-β-gal) activity.

Cellular senescence was determined using the “Senescence beta-Galactosidase Staining Kit” (Cell Signaling Technology, Danvers, MA, USA) according to the manufacturer’s instructions. As a positive control, cells were treated for 48 h with 100 μM of the topoisomerase II inhibitor etoposide (R&D Systems, Minneapolis, MN, USA). For analysis, 4-7 images per 6-well were acquired on the fluorescence microscope “IX83” (Olympus K.K., Shinjuku, Tokyo, Japan) in brightfield.

Cell cycle analysis

For an analysis of the cell cycle, cells were fixed and permeabilized with cold 70 % methanol overnight at 4 °C. Subsequently, the cells were diluted in 0.2 % BSA in PBS, treated with 0.1 μg/μl RNAse A at 37 °C for 30 min and stained with propidium iodide (PI) (PromoKine, PromoCell GmbH, Heidelberg, Germany) corresponding to the cell number (for 500,000 cells 25 μl of a 1 mg/ml solution) on ice for 30 min. The intensity of the PI signal per cell was measured using the flow cytometer “FACSCalibur^{TM}” (Becton, Dickinson and Company, Franklin Lakes, NJ, USA). The data were analyzed using “FlowJo_v10.8.0” (Becton, Dickinson and Company, Franklin Lakes, NJ, USA). Cell gates were set the same for one cell line. Single cell gates were set as appropriate and were kept the same for associated samples in each case. For analysis of cell cycle phases, the “Dean-Jett-Fox” model was used. Gating and analysis of SubG1 cells was performed using the “FACSCalibur^{TM}” software (Becton, Dickinson and Company, Franklin Lakes, NJ, USA).

Analysis of apoptosis

Apoptosis in the melanoma cells was detected with annexin V-fluorescein isothiocyanate (FITC)- and PI-staining of living cells using the "Annexin V-FITC Detection Kit" (PromoKine, PromoCell GmbH, Heidelberg, Germany) according to the manufacturer’s instructions. Analysis was done with the flow cytometer “FACSCaliburTM” (Becton, Dickinson and Company,

Franklin Lakes, NJ, USA) and the software "FlowJo_v10.8.0" (Becton, Dickinson and Company, Franklin Lakes, NJ, USA). Cell gates were set the same for each cell line, quadrant gates were kept the same for associated samples.

CRISPR/Cas9-mediated knockout of AGO2 in melanoma cells

For the "clustered regularly interspaced short palindromic repeats" (CRISPR)/Cas9-mediated knockout of AGO2 two plasmids were used described previously by van Eijl et al. [1]. The plasmids containing the guide RNAs and Cas9 were subcloned using EcoRV-HF and EcoRI-HF into the pU6-(BbsI)_CBh-Cas9-T2A-mCherry vector which was a gift from Ralf Kuehn (Addgene plasmid #64324; http://n2t.net/addgene:64324; RRID:Addgene_64324) [2]. The two guide RNA sequences are complementary to an intronic region upstream of exon 2 of the AGO2 gene and to the beginning of exon 2 resulting in a 58 base pair genomic deletion preventing exon 2 from being incorporated into the AGO2 mRNA. Mel Im and Mel Juso cells were transfected with 2µg of each plasmid and after 24 h red fluorescent cells were sorted to single cells using a "FACS Aria II SORP" cell sorter (Becton, Dickinson and Company, Franklin Lakes, NJ, USA). To verify a successful homozygous knockdown, genomic DNA (gDNA) was isolated from the single cell derived clones using the "QIAamp DNA Mini Kit" (QIAGEN GmbH, Hilden, Germany) according to the manufacturer's instructions. GDNA was amplified via PCR and sequenced.

RNA isolation, DNase I or RNase R digestion and reverse transcription

For RNA isolation of melanoma cells or tissue samples, the "E.Z.N.A Total RNA Kit I" (Omega Bio-Tek, VWR, Germany) was used and the protocol was performed according to the manufacturer's instructions. For DNase digestion during RNA isolation, the "DNase I Digestion Set" (Omega Bio-Tek, VWR, Germany) was used. RNA concentration was determined on a "Nanodrop 200c" spectrophotometer (PeqLab, Thermo Fischer Scientific, Waltham, MA, USA).

For digestion with RNase R (Epicentre, Illumina Inc., Madison, WI, USA) 5 µg of RNA were incubated with or without 2.5 µl RNase R from a 1:4 dilution (5 U/µl) in a total volume of 20 µl for 15 min at 37 °C. Subsequently, 180 µl of RNase-free H_2O was added and the RNA was purified by using 200 µl of a phenol/chloroform/isoamyl alcohol solution pH 4.5 (125:24:1, Ambion™, Invitrogen, Thermo Fischer Scientific, Waltham, MA, USA) and precipitated with an ethanol-sodium acetate solution (3M, pH 5.2), and 1 µl carrier RNA (100 ng/µl) for 20 min at -80 °C.

Reverse transcription was performed with 1/5 volume of RNase R-digested RNA or 500 ng total RNA using the "SuperScript® II Reverse Transcriptase Kit" and a random p(dN)6 primer

(Roche Diagnostics GmbH, Mannheim, Germany) according to the manufacturer's instructions. Remaining RNA was digested with 1 µl RNase A (10 µg/µl solution, Roche Diagnostics GmbH, Mannheim, Germany) for 30 min at 37 °C. For reverse transcription with a poly-adenine specific primer, 4 µg RNA and 2 µl of the primer (CCAGTGAGCAGAGTGACGAG-GACTCGAGCTCAAGCTTTTTTTTTTTTTTTTT, 25 µM) was used.

Polymerase chain reaction (PCR)

For PCR the" FastStart™ Taq DNA Polymerase (5 U/µl)"-kit (Roche Diagnostics GmbH, Mannheim, Germany) was used in the following reaction: 1 µl cDNA (or 5 µl gDNA), 0.5 µl forward and reverse primer (20 µM) (Table 1), 0.5 µl "FastStart™ Taq" polymerase, 0.5 µl dNTPs (10 mM each), 2 µl PCR reaction buffer with 20 mM $MgCl_2$ and 15 µl (or 11 µl) H_2O. Amplification was performed with 5 min at 95 °C, 36 cycles of: 15 s at 95 °C, 30 s annealing at a primer-specific temperature (table 1), 30 s per 500 bp at 72 °C and one final step at 72 °C for 5-10 min. PCR products were mixed with loading buffer (0.25 % bromophenol blue, 0.25 % xylene cyanol, 40 % sucrose) and analyzed on a gel with 1.5-3 % agarose (Biozym Scientific GmbH, Hessisch Oldendorf, Germany) in TAE buffer (40 mM Trizma® base (Sigma-Aldrich Chemie GmbH, Steinheim, Germany), 0.1 % acetic acid and 1 mM EDTA, pH 8.0) with 5 % "Midori Green Advance" (Biozym Scientific GmbH, Hessisch Oldendorf, Germany). For size comparison the GeneRuler 100bp or 1kb plus DNA Ladder (Thermo Fischer Scientific, Waltham, MA, USA) was used. For sequencing, elution of the DNA was performed using the "QIAquick® Gel Extraction Kit" (QIAGEN GmbH, Hilden, Germany) according to the manufacturer's instructions. DNA sequencing was carried out by the Microsynth Seqlab GmbH (Göttingen, Germany). Sequence alignment was performed using the software "GENtle" (http://gentle.magnusmanske.de) and the AGO2 reference sequence NM_012154.5 (https://www.ncbi.nlm.nih.gov/refseq).

Table 1: Primer used for PCR

PCR	Primer name	Sequence	Annealing temperature (°C)
Exon1-3	hAGO2-mRNA-42-fwd	ATGTACTCGGGAGCCG	59
	hAGO2-mRNA-344-rev	GTATAGATTCTTCCTGCCGTC	
Exon1/Exon3-Exon4	hAGO2-Ex1/3-fwd	GCCCCGGGAAATCG	60
	hAGO2-mRNA-524-rev	GGCCTGGATCGTCTCAAAG	
HIPK3 lin [3]	HIPK3_linE3-4_fwd	TATTGGGGTTGCCATTTTGT	58
	HIPK3_linE3-4_rev	GTTCTCCTGGCAAACCTTGA	
HIPK3 circ [3]	HIPK3_circE2_fwd	TCGGCCAGTCATGTATCAAA	58
	HIPK3_circE2_rev	CCCTTAGTGGGAGGATGAGA	
Exon1/3-Exon3	hAGO2-Ex1/3-fwd	GCCCCGGGAAATCG	60
	hAGO2-mRNA-344-rev	GTATAGATTCTTCCTGCCGTC	
Exon1/3-6	hAGO2-Ex1/3-fwd	GCCCCGGGAAATCG	60
	hAGO2-mRNA-735-rev	CGATTACTGGCTGTGCCTTG	
Exon1/3-7	hAGO2-Ex1/3-fwd	GCCCCGGGAAATCG	60
	hAGO2-mRNA-892-rev	ACATTGCAGACGCGGTACTT	

Exon1/3-15	hAGO2-Ex1/3-fwd	GCCCCGGGAAATCG	60
	hAGO2-mRNA-2011-rev	GTGGACTTGTAGAACTGGATG	
Exon1/3-19	hAGO2-Ex1/3-fwd	GCCCCGGGAAATCG	60
	hAGO2-mRNA-2556-rev	GGTCTCGCCCGTTACTCTG	
Exon1/3-3'-UTR	hAGO2-Ex1/3-fwd	GCCCCGGGAAATCG	60
	hAGO2-mRNA-3498-2rev	CTGCAAACCAGATATATATATTCTT-CTCTTACATTAAA	
AGO2 genomic [1]	hAgo2 genom 49929 fwd	GACACTTTTGGGCTCTTTGC	60
	hAgo2 genom 50424 rev	AACTCTCCTCGGGCACTTCT	

Quantitative real-time PCR (qRT-PCR)

The qRT-PCR was performed in "LightCycler® 480 Multiwell plates 96 white" (Roche Diagnostics GmbH, Mannheim, Germany) using a "LightCycler® 480 II" device (Roche Diagnostics GmbH, Mannheim, Germany) and the following program: 10 min at 95 °C (4.4 °C/s), 45 cycles with 10 s at 95 °C (4.4 °C/s), 10 s at 60 °C (2.2 °C/s) and 20 s at 72 °C (4.4 °C/s), measurement of the created fluorescence after each cycle at a primer specific temperature (table 2), a melting curve with 5 s at 95 °C (4.4 °C/s), 1 min at 65°C (2.2 °C/s) and heating to 97 °C with 0.11 °C/s and 5 measurements per °C and a final cooling step for 30 s to 40°C (2.2 °C/s). For each cDNA two replicate wells, each with 1µl cDNA, 10 µl "LightCycler® 480 SYBR Green I Master" (Roche Diagnostics GmbH, Mannheim, Germany), 0.5 µl forward and reverse primer (20 µM) and 8 µl H_2O were prepared.

Relative expression of a gene was calculated using the ΔCP method, efficiency$^{CP\ housekeeper}$ divided through efficiency$^{CP\ gene}$, where the efficiency was determined for each primer pair using a dilution series of the cDNA and the "AbsQuant/FitPoints" method of the "Light Cycler® 480 II".

Table 2: Primer used for qRT-PCR

**Gene**	**Primer name**	**Sequence**	**Temperature of measurement (≤ °C)**	**Efficiency**
AGO2 (exon 17-19)	hAGO2-mRNA-2283-fwd	TTCTACCTGTGTAGTCACGCTG	86	1.92
	hAGO2-mRNA-2556-rev	GGTCTCGCCCGTTACTCTG		
AGO2 full length (exon1/2)	hAgo2-mRNA-Exon1/2 fwd	GCCCCGCACTTGCAC	84	1.95
	hAGO2-mRNA-354-rev	GCATGGCTGTGTATAGATTCTTCC		
AGO2-ex1/3	hAGO2-Ex1/3-fwd	GCCCCGGGAAATCG	85	1.85
	hAGO2-mRNA-524-rev	GGCCTGGATCGTCTCAAAG		
β-actin	Beta-Aktin_cyto for	CTACGTCGCCCTGGACTTCGAGC	86	2.03
	Beta-Aktin_cyto rev	GATGGAGCCGCCGATCCACACGG		
GFP	eGFP 67 fwd	CGTAAACGGCCACAAGTTCA	88	1.78
	eGFP 241 rev	CTTCATGTGGTCGGGGTAGC		

MiRNA isolation and qRT-PCR for miRNA expression

For isolation of miRNAs from melanoma cells the “miRNeasy Mini Kit” (Qiagen, Hilden, Germany) and for reverse transcription of 200 ng miRNA the “miRCURY LNA RT Kit” (Qiagen, Hilden, Germany) were used according to the manufacturer’s instructions. QRT-PCR was performed with the “miRCURY LNA SYBR® Green PCR Kit” (Qiagen, Hilden, Germany) and the “miRCURY LNA miRNA PCR Assays” for hsa-miR-105-5p (YP00204389), hsa-miR-25-3p (YP00204361) and has-miR-192-5p (YP00204534). As housekeeper the U6 snRNA (YP02119464) was used. For the PCR reaction the mi-cDNA was diluted 1:60 with RNase free water and 1 µl were used in a 10 µl reaction. The qRT-PCR was performed in “LightCycler® 480 Multiwell plates 96 white” (Roche Diagnostics GmbH, Mannheim, Germany) using a “LightCycler® 480 II” device (Roche Diagnostics GmbH, Mannheim, Germany).

Cloning of pAGO2-ex1/3HAint and pAGO2-ex1/3GFP

For cloning of an AGO2-ex1/3 expression plasmid, exon2 of the AGO2 sequence was removed from pIRESneo-FLAG/HA-AGO2 [4] using the “Phusion Site-Directed Mutagenesis Kit” (Thermo Fischer Scientific, Waltham, MA, USA) according to the manufacturer's instructions and the following 5’-phosphorylated primers: hAGO2-ex3-fwd P-GGAAATCGTG-GAACACATGGTCCAG, hAGO2-ex1-rev P-CGGGGCCGGCTCCC.

Subsequently, for adding an C-terminal GFP tag to the AGO2-ex1/3 sequence replacing the stop codon of the first reading frame (pAGO2-ex1/3GFP), the sequence of the described AGO2-ex1/3 plasmid was amplified using the “Phusion Hot Start II High-Fidelity DNA Polymerase (2 U/µL)” (Thermo Fischer Scientific, Waltham, MA, USA) according to the manual with the following primers containing recognition sites for restriction enzymes marked in bold: hAGO2-ex1/3-HindIII-fwd TAC**AAGCTT**ATGTACTCGGGAGCCGG, hAGO2-ex1/3-NotI-rev TTA**GCGGCCGC**CCGCAGGACACCCACTTGA. The PCR product was cloned into the pcDNA3.1+C-eGFP plasmid (GenScript, Leiden, The Netherlands).

The pGFP control plasmid was cloned from pAGO2-ex1/3GFP via digestion with HindIII-HF and NotI-HF and subsequent incubation of 1 µg cut plasmid with 0.3 µl of the “DNA Polymerase I, Large (Klenow) Fragment” (New England BioLabs Inc., Ipswich, MA, USA), 6.6 µl dNTPs (see chapter “PCR”, 1:100 dilution) 2 µl T4 DNA ligase buffer (New England BioLabs Inc., Ipswich, MA, USA) and 0.24 µl H_2O for 15 min at 25 °C. The reaction was stopped with 5 µl EDTA (50 mM solution) for 20 min at 75 °C.

The pAGO2-ex1/3HAint plasmid with an internal HA tag was cloned using mutagenesis by overlap extension according to Ho et al. 1989 [5] with the following primers A-D containing recognition sites for restriction enzymes and the sequence of the HA-tag marked in bold: hAGO2-ex1/3-EcoRI-fwd-A AC**GAATTC**ATGTACTCGGGAGCCGG, hAGO2-ex1/3-rev-B

CAGAGGTTTTTGTTG**AGCGTAATCGGGCACGTCATAAGGGTA**TTCTTCAATACTTTTAA, hAGO2-ex1/3-fwd-C TTAAAAGTATTGAAGAA**TACCCTTATGACGTGCCCGATTAGCT**CAA-CAAAAACCTCTG, hAGO2-ex1/3-XbaI-rev-D GAC**TCTAGA**TCAAGCAAAGTACATGGTG-CGC. Two PCR reactions were performed using the "Phusion Hot Start II High-Fidelity DNA Polymerase (2 U/µL)" (Thermo Fischer Scientific, Waltham, MA, USA) according to the manual with the primers A+B and C+D, respectively and the AGO2-ex1/3 plasmid described above as template. The resulting products were purified via an agarose gel. 1µl of each eluted product was used as template for the next PCR using the primers A+D and a two-step PCR program was performed with 30s 98°C, 32 cycles with 15s 98°C and 100s 72 °C and a final elongation of 15min at 72°C. The resulting PCR product was digested using EcoRI-HF and XbaI and cloned into pcDNA3.1(+) (GeneArt, Thermo Fischer Scientific, Waltham, MA, USA). Cloning resulted in a plasmid containing the AGO2-ex1/3-sequence and an HA-tag after A735 (between E245/Q246) in the AGO2-CDS.

Western blot

Cells were lysed with radioimmunoprecipitation-assay(RIPA)-buffer (150 mM NaCl, 0.5 % sodium deoxycholate, 1 % "Nonidet® P40", "cOmplete™" protease inhibitor tablets (Roche Diagnostics, Mannheim, Germany), 0.1 % sodium dodecyl sulfate (SDS), 50 mM "Trizma® hydrochloride" (Sigma-Aldrich Chemie GmbH, Steinheim, Germany) solution pH 7.5) and the protein concentration was determined using the "Pierce™ BCA Protein Assay Kit" (Thermo Fischer Scientific, Waltham, MA, USA) in duplicates according to the manufacturer's instructions. 40 µg of protein lysate were separated on a 8.75-15 % SDS-polyacrylamide gel and blotted on an "Amersham™ Hybond™ 0,2 µm" polyvinylidene difluoride membrane (GE Healthcare Life Science Europe GmbH, Freiburg, Germany) in a semi dry process. The following antibodies were used for staining: AGO2 N-terminal epitope (clone 11A9 [6], hybridoma supernatant diluted 1:10 in 5% milk powder (Carl Roth GmbH & Co.KG, Karlsruhe, Germany) in TBS-T (50 mM "Trizma® base" solution pH 7.6, 150 mM NaCl, 1% "Tween® 20"), AGO2 C-terminal epitope (rabbit polyclonal, ab32381 (abcam, Cambridge, UK) 1:1000 in 5% milk powder in TBS-T), HA (clone 16B12 (Biolegend, San Diego, CA, USA), 1:1000 in 5% milk powder in TBS-T), GFP (rabbit polyclonal, A-11122 (Thermo Fischer Scientific, Waltham, MA, USA), 1:2000 in 5% BSA in TBS-T), β-actin (clone AC-15 (Sigma-Aldrich Chemie GmbH, Steinheim, Germany), 1:5000 in PBS) with the respective secondary antibodies anti-rat-alkaline phosphatase (AP) (Carl Roth GmbH & Co.KG, Karlsruhe, Germany, 1:4000 in TBS-T) or anti-mouse-, rat-, or rabbit-horseradish peroxidase (HRP) (Cell Signaling Technology, Danvers, MA, USA, 1:3000 in TBS-T)) and developed using the "Clarity™ Western ECL Substrate Kit" (Bio-Rad, Hercules, CA, USA) for HRP or the „BCIP/NBT Kit" (Life Technologies, Frederick, MD, USA) for AP according to

the manual. The HRP signal was detected using the „Intas ECL Chemocam“ gel documentation system (Intas Science Imaging Instruments GmbH, Göttingen, Germany).

Molecular structure and dynamics simulations

A high-resolution X-ray structure of human Argonaute 2 bound to a target RNA (PDB code: 4z4d; [7]) was chosen as basis for the molecular dynamics (MD) simulations. Since the focus of the MD simulations was on the free protein, the RNA was removed from the simulation setup. Four missing loops (amino acid 121-126, 270-275, 296-304, and 822-835) of the protein structure were built with via Modeller 9.25 [8–10] using the ModLoop procedure. An N-terminal acetyl group was added to the resulting model, to account for the missing N-terminal residues 1-21 in the PDB file.

The structure of the truncated variant AGO2-ex1/3, which lacks the first N-terminal 77 residues, was prepared by deleting the respective residues from the full length model. For both models, AGO2 and AGO2-ex1/3, two slightly different conformations were created using the alternative side chain conformations present in the PDB file, to ensure independent simulation runs for a better sampling. Histidine protonation states were chosen according to propka [11] from the pdb2pqr server [12]. All systems were solvated in a capped octahedral TIP3P [13] water box with at least 15 Å to the periodic bounding box border and electrically neutralized by the addition of chloride counter ions.

Following our established simulation procedure [14], a three-step geometry optimization was performed first, then the systems were heated to 310 K over the course of 2 ns, and subsequently simulated for 1.5 µs with a time step of 2 fs in an NPT ensemble with 1 bar. Amber 20 [15] with the parm14SB [16] parameter set was used for all simulations; all production runs were performed on GPUs [17, 18].

To enhance conformational sampling, all four optimized systems were additionally investigated by Gaussian-accelerated MD (GaMD) [19]. For this purpose, 10 ns of conventional MD were performed first, followed by 20 ns of boosted MD with statistics, and finally by 1.5 µs production runs.

Root mean square deviations (RMSD) of the protein backbone atoms with respect to the initial structure were calculated via cpptraj from AmberTools21 [20]. Visualization and graphical representations were created with VMD [21], graphs from the simulations were created with gnuplot.

From the eight simulations conducted in total, the graphs and analyses are shown for the GaMD runs in the supplementary file 2. The four conventional MD simulations showed very similar results with a slightly smaller overall dynamics, which is due to the less intense sampling compared to the GaMD.

RNA-Seq library preparation, mapping, visualization and data analysis

RNA-Seq analysis was performed with two two (Mel Im, Mel Wei) or three independent biological replicates (Mel Juso treated with siAGO2-ex1/3 or siCtrl respectively). RNA was isolated as described above. Library preparation was performed using the "TruSeq® Stranded Total RNA Library Prep Human/Mouse/Rat Kit" according to the manufacturer's instructions (Illumina Inc., San Diego, CA, USA). The resulting libraries were checked for size (200-500 bp) by TapeStation 4200 (Agilent, Santa Clara, CA, USA) using the High-Sensitivity DNA Kit (Agilent, Santa Clara, CA, USA) and concentration by the Qubit 4 Fluorometer (Thermo Fischer Scientific, Waltham, MA, USA). The samples were sequenced on a HiSeq4000 with a paired-end module (Illumina, Inc., San Diego, CA, USA) according to the paired-end RNA sequencing protocols from Illumina. Sequencing was performed from each side of a fragment about 75 bp long with a mean number of 20 million reads per sample. After quality check using FastQC (https://www.bioinformatics.babraham.ac.uk/projects/fastqc/), paired-end reads were aligned to the human reference genome (hg38) using the STAR alignment software (v 2.5.2a) [22]. After mapping, only reads that mapped to a single unique location were considered for further analysis. The mapped reads were then used to generate a count table using the feature-counts software (v 1.4.6-p5) [23]. The raw reads were filtered, normalized, and visualized by using by using R (version 4.0.1) or the Integrative Genomics Viewer (https://software.broadinstitute.org/software/igv/, [24]).

DESeq2 package version 1.26.0 [25] was used for logarithmic transformation of the data and for data exploration. Differential expression analysis was done using the DESeq2 standard approach. Adjusted p-values are calculated using the Benjamini–Hochberg method within DESeq2. Gene annotations were added to the result files using Ensemble data [26].

Gene set enrichment analysis (GSEA) was performed using the GSEA software version 4.0.3 (https://www.gsea-msigdb.org/gsea/index.jsp, [27]) and gene set collections from the "Molecular Signature Database" (MSigDB version 7.2, https://www.gsea-msigdb.org/gsea/msigdb/index.jsp). For the analysis, gene set permutation was performed with 1000 permutations and "Signal2Noise" metric. The "STRING" network analysis was conducted with https://string-db.org (version 11.5 Aug. 2021 [28]), with an minimum required interaction score of medium confidence (0.400) and formatted using the stringApp in Cytoscape (version 3.8.2 [29]). Results of the differential expression analysis and the GSEA analysis were visualized using "RStudio" (version 1.3.1093) with the "ggplot2" package from the "tidyverse 1.3.0", the "ggrepel" and the "ggforce" package. The volcano plot was created using the functions ggplot, geom_point, scale_color_manual, theme_mininal and geom_text_repel. The violin scatter plot was created with ggplot, geom_sina and theme_mininal.

Data for miRNAs differentially expressed in melanoma cell lines compared to NHEM was published before [30] and obtained from the Gene Expression Omnibus database (accession number GSE174334). The heatmap with normalized reads contains miRNAs which are expressed in melanoma and/or NHEM (at least in two samples >10 normalized reads) as well as significantly upregulated in melanoma cell lines derived from primary tumor (Mel Juso, Mel Ei, Mel Wei) compared to NHEM (log2fold change ≥0.9, p-value<0.05 [30]) and whose target genes are simultaneously significantly enriched in siAGO2-ex1/3-transfected Mel Juso cells (p-value <0.05, false discovery rate (FDR) < 0.25, obtained from GSEA analysis described above). The heatmap was created with Microsoft Excel using conditional formatting with graduated color scale for each line.

References for the additional material and methods part

1. Van Eijl RAPM, Van Den Brand T, Nguyen LN, Mulder KW (2017) Reactivity of human AGO2 monoclonal antibody 11A9 with the SWI/SNF complex: A case study for rigorously defining antibody selectivity. Sci Rep 7:1–11. https://doi.org/10.1038/s41598-017-07539-4
2. Chu VT, Weber T, Wefers B, et al (2015) Increasing the efficiency of homology-directed repair for CRISPR-Cas9-induced precise gene editing in mammalian cells. Nat Biotechnol 33:543–548. https://doi.org/10.1038/nbt.3198
3. Starke S, Jost I, Rossbach O, et al (2015) Exon Circularization Requires Canonical Splice Signals. Cell Rep 10:103–111. https://doi.org/10.1016/J.CELREP.2014.12.002
4. Meister G, Landthaler M, Patkaniowska A, et al (2004) Human Argonaute2 mediates RNA cleavage targeted by miRNAs and siRNAs. Mol Cell 15:185–197. https://doi.org/10.1016/j.molcel.2004.07.007
5. Ho SN, Hunt HD, Horton RM, et al (1989) Site-directed mutagenesis by overlap extension using the polymerase chain reaction. Gene 77:51–59. https://doi.org/10.1016/0378-1119(89)90358-2
6. Rüdel S, Flatley A, Weinmann L, et al (2008) A multifunctional human Argonaute2-specific monoclonal antibody. RNA 14:1244–1253. https://doi.org/10.1261/rna.973808
7. Schirle NT, Sheu-Gruttadauria J, Chandradoss SD, et al (2015) Water-mediated recognition of t1-adenosine anchors Argonaute2 to microRNA targets. Elife 4:e07646. https://doi.org/10.7554/eLife.07646
8. Fiser A, Do RKG, Šali A (2000) Modeling of loops in protein structures. Protein Sci 9:1753–1773. https://doi.org/10.1110/PS.9.9.1753
9. Martí-Renom MA, Stuart AC, Fiser A, et al (2000) Comparative protein structure modeling of genes and genomes. Annu Rev Biophys Biomol Struct 29:291–325. https://doi.org/10.1146/ANNUREV.BIOPHYS.29.1.291
10. Šali A, Blundell TL (1993) Comparative protein modelling by satisfaction of spatial restraints. J Mol Biol 234:779–815. https://doi.org/10.1006/JMBI.1993.1626
11. Li H, Robertson AD, Jensen JH (2005) Very fast empirical prediction and rationalization of protein pKa values. Proteins 61:704–721. https://doi.org/10.1002/PROT.20660

12. Jurrus E, Engel D, Star K, et al (2018) Improvements to the APBS biomolecular solvation software suite. Protein Sci 27:112–128. https://doi.org/10.1002/PRO.3280
13. Jorgensen WL, Chandrasekhar J, Madura JD, et al (1998) Comparison of simple potential functions for simulating liquid water. J Chem Phys 79:926. https://doi.org/10.1063/1.445869
14. Söldner CA, Sticht H, Horn AHC (2017) Role of the N-terminus for the stability of an amyloid-β fibril with three-fold symmetry. PLoS One 12: e0186347. https://doi.org/10.1371/JOURNAL.PONE.0186347
15. Case DA, Aktulga HM, Belfon K, et al (2021) Amber 2021. University of Califormia, San Francisco. https://ambermd.org/doc12/Amber21.pdf
16. Maier JA, Martinez C, Kasavajhala K, et al (2015) ff14SB: Improving the Accuracy of Protein Side Chain and Backbone Parameters from ff99SB. J Chem Theory Comput 11:3696–3713. https://doi.org/10.1021/ACS.JCTC.5B00255
17. Götz AW, Williamson MJ, Xu D, et al (2012) Routine Microsecond Molecular Dynamics Simulations with AMBER on GPUs. 1. Generalized Born. J Chem Theory Comput 8:1542–1555. https://doi.org/10.1021/CT200909J
18. Salomon-Ferrer R, Götz AW, Poole D, et al (2013) Routine Microsecond Molecular Dynamics Simulations with AMBER on GPUs. 2. Explicit Solvent Particle Mesh Ewald. J Chem Theory Comput 9:3878–3888. https://doi.org/10.1021/CT400314Y
19. Miao Y, Feher VA, McCammon JA (2015) Gaussian Accelerated Molecular Dynamics: Unconstrained Enhanced Sampling and Free Energy Calculation. J Chem Theory Comput 11:3584–3595. https://doi.org/10.1021/ACS.JCTC.5B00436
20. Roe DR, Cheatham TE (2013) PTRAJ and CPPTRAJ: Software for Processing and Analysis of Molecular Dynamics Trajectory Data. J Chem Theory Comput 9:3084–3095. https://doi.org/10.1021/CT400341P
21. Humphrey W, Dalke A, Schulten K (1996) VMD: visual molecular dynamics. J Mol Graph 14:33–38. https://doi.org/10.1016/0263-7855(96)00018-5
22. Dobin A, Davis CA, Schlesinger F, et al (2013) STAR: Ultrafast universal RNA-seq aligner. Bioinformatics 29:15–21. https://doi.org/10.1093/bioinformatics/bts635
23. Liao Y, Smyth GK, Shi W (2014) FeatureCounts: An efficient general purpose program for assigning sequence reads to genomic features. Bioinformatics 30:923–930. https://doi.org/10.1093/bioinformatics/btt656
24. Thorvaldsdóttir H, Robinson JT, Mesirov JP (2013) Integrative Genomics Viewer (IGV): High-performance genomics data visualization and exploration. Brief Bioinform 14:178–192. https://doi.org/10.1093/bib/bbs017
25. Love MI, Huber W, Anders S (2014) Moderated estimation of fold change and dispersion for RNA-seq data with DESeq2. Genome Biol 15:550. https://doi.org/10.1186/s13059-014-0550-8
26. Howe KL, Achuthan P, Allen J, et al (2021) Ensembl 2021. Nucleic Acids Res 49:D884–D891. https://doi.org/10.1093/nar/gkaa942
27. Subramanian A, Tamayo P, Mootha VK, et al (2005) Gene set enrichment analysis: A knowledge-based approach for interpreting genome-wide expression profiles. Proc Natl Acad Sci U S A 102:15545–15550. https://doi.org/10.1073/pnas.0506580102

28. Szklarczyk D, Gable AL, Nastou KC, et al (2021) The STRING database in 2021: Customizable protein-protein networks, and functional characterization of user-uploaded gene/measurement sets. Nucleic Acids Res 49:D605–D612. https://doi.org/10.1093/nar/gkaa1074

29. Shannon P, Markiel A, Ozier O, et al (2003) Cytoscape: A software Environment for integrated models of biomolecular interaction networks. Genome Res 13:2498–2504. https://doi.org/10.1101/gr.1239303

30. Linck-Paulus L, Lämmerhirt L, Völler D, et al (2021) Learning from Embryogenesis—A Comparative Expression Analysis in Melanoblast Differentiation and Tumorigenesis Reveals miRNAs Driving Melanoma Development. J Clin Med 10:2259. https://doi.org/10.3390/jcm10112259
